# Mitochondrial related variants associated with cardiovascular traits

**DOI:** 10.3389/fphys.2024.1395371

**Published:** 2024-08-27

**Authors:** Marisa Cañadas-Garre, Joaquín J. Maqueda, Blanca Baños-Jaime, Claire Hill, Ryan Skelly, Ruaidhri Cappa, Eoin Brennan, Ross Doyle, Catherine Godson, Alexander P. Maxwell, Amy Jayne McKnight

**Affiliations:** ^1^ Molecular Epidemiology and Public Health Research Group, Centre for Public Health, Queen’s University Belfast, Institute for Clinical Sciences A, Royal Victoria Hospital, Belfast, United Kingdom; ^2^ MRC Integrative Epidemiology Unit, Bristol Medical School (Population Health Sciences), University of Bristol Oakfield House, Belfast, United Kingdom; ^3^ Laboratory of Experimental Oncology, IRCCS Istituto Ortopedico Rizzoli, Bologna, Italy; ^4^ Department of Experimental, Diagnostic and Specialty Medicine (DIMES), University of Bologna, Bologna, Italy; ^5^ Instituto de Investigaciones Químicas (IIQ), Centro de Investigaciones Científicas Isla de la Cartuja (cicCartuja), Universidad de Sevilla, Consejo Superior de Investigaciones Científicas (CSIC), Sevilla, Spain; ^6^ UCD Diabetes Complications Research Centre, Conway Institute of Biomolecular and Biomedical Research, University College Dublin, Dublin, Ireland; ^7^ School of Medicine, University College Dublin, Dublin, Ireland; ^8^ Mater Misericordiae University Hospital, Dublin, Ireland; ^9^ Regional Nephrology Unit, Belfast City Hospital Belfast, Belfast, United Kingdom

**Keywords:** cardiovascular disease, coronary artery disease, mitochondrial DNA, blood pressure, hypertension, diabetes, UK Biobank, mitochondrial haplogroups

## Abstract

**Introduction:**

Cardiovascular disease (CVD) is responsible for over 30% of mortality worldwide. CVD arises from the complex influence of molecular, clinical, social, and environmental factors. Despite the growing number of autosomal genetic variants contributing to CVD, the cause of most CVDs is still unclear. Mitochondria are crucial in the pathophysiology, development and progression of CVDs; the impact of mitochondrial DNA (mtDNA) variants and mitochondrial haplogroups in the context of CVD has recently been highlighted.

**Aims:**

We investigated the role of genetic variants in both mtDNA and nuclear-encoded mitochondrial genes (NEMG) in CVD, including coronary artery disease (CAD), hypertension, and serum lipids in the UK Biobank, with sub-group analysis for diabetes.

**Methods:**

We investigated 371,542 variants in 2,527 NEMG, along with 192 variants in 32 mitochondrial genes in 381,994 participants of the UK Biobank, stratifying by presence of diabetes.

**Results:**

Mitochondrial variants showed associations with CVD, hypertension, and serum lipids. Mitochondrial haplogroup J was associated with CAD and serum lipids, whereas mitochondrial haplogroups T and U were associated with CVD. Among NEMG, variants within Nitric Oxide Synthase 3 (*NOS3*) showed associations with CVD, CAD, hypertension, as well as diastolic and systolic blood pressure. We also identified Translocase Of Outer Mitochondrial Membrane 40 (*TOMM40*) variants associated with CAD; Solute carrier family 22 member 2 (*SLC22A2*) variants associated with CAD and CVD; and *HLA-DQA1* variants associated with hypertension. Variants within these three genes were also associated with serum lipids.

**Conclusion:**

Our study demonstrates the relevance of mitochondrial related variants in the context of CVD. We have linked mitochondrial haplogroup U to CVD, confirmed association of mitochondrial haplogroups J and T with CVD and proposed new markers of hypertension and serum lipids in the context of diabetes. We have also evidenced connections between the etiological pathways underlying CVDs, blood pressure and serum lipids, placing *NOS3*, *SLC22A2*, *TOMM40* and *HLA-DQA1* genes as common nexuses.

## Introduction

Cardiovascular disease (CVD) is an umbrella term that encompasses all diseases of the heart and blood vessels, including heart disease (involving the heart) and vascular disease (involving the blood vessels) (Lopez et al., 2023). Coronary artery disease (CAD), sometimes called coronary heart disease or ischemic heart disease, is the most common type of heart disease and is characterized by a narrowing or blockage of the coronary arteries (Coronary Artery Disease, 2023; Coronary Artery Disease (CAD): Symptoms & Treatment, n.d.). CVD is the major cause of deaths worldwide, accounting for more than 30% of mortality (WHO, 2023; Tsao et al., 2023). CAD is the leading cause of death, accounting for 16% of global mortality in 2019 (WHO, 2023). Diabetes is a major risk factor for development of CVD and for people with diabetes, CVD represents the leading cause of morbidity and mortality (WB and DL, 1979). Individuals with type 2 diabetes mellitus (T2DM) have a 2–4 times increased risk of CVD (Kishore et al., 2012; Visseren et al., 2021; Yang et al., 2021).

CVD is a complex multifactorial condition arising from the combined influence of environmental and hereditary factors. Well established modifiable risk factors for CVDs include hypertension, diabetes, hypercholesterolemia and smoking with these factors used in the estimation of the 10-year risk of incident cardiovascular events (Goff et al., 2014; Cadby et al., 2020). Serum lipids, namely, total cholesterol (Chol), low-density lipoprotein (LDL), high-density lipoprotein cholesterol (HDL) and triglycerides (TG) are directly implicated in the development of CVDs, and are used as risk factors to predict long-term CVD risk and adverse clinical outcomes (Goff et al., 2014; Cadby et al., 2020), and as therapeutic targets for CVDs (Ference et al., 2017; Echouffo-Tcheugui et al., 2020). The aetiology of CVD is clearly influenced by genetics, as evidenced in many studies (Marino and Digilio, 2000; Muntean et al., 2017; Silva et al., 2023; Safdar et al., 2024). CVD, and especially CAD, show polygenic architecture and a substantial heritability, estimated between 40% and 60% (Watkins and Farrall, 2006; Dai et al., 2016; Khera and Kathiresan, 2017; Inouye et al., 2018a; Drobni et al., 2022). Genome-wide association studies (GWAS) have identified associations between single nucleotide polymorphisms (SNPs) and CAD, myocardial infarction, and other CVDs (Companioni et al., 2011; Muntean et al., 2017; Silva et al., 2023). A recent systematic review confirmed at least 71 genetic variants as susceptibility loci for CAD (Silva et al., 2023). Beyond SNPs, there are other genetic causes of CVD, including chromosomal aberrations, copy number variations and epigenomics (Liu et al., 2023; Safdar et al., 2024). However, despite the growing number of hereditary factors contributing to CVD, the cause of the vast majority of CVDs remains unclear (Safdar et al., 2024). Mitochondria play a significant role in the pathophysiology, development and progression of CVDs, through key mechanisms such as excessive reactive oxygen species production, mitochondrial dysfunction, and genetic factors as mitochondrial DNA (mtDNA) damage or mutations (Venter et al., 2018; Siasos et al., 2018; Poznyak et al., 2020; Lin et al., 2021; Calabrese et al., 2022; Yang et al., 2022). In cardiac mitochondria, mtDNA is important in the mitochondrial life circle and the proper functioning of oxidative phosphorylation (OXPHOS). Irreversible mtDNA damage leads to mtDNA mutations, which in turn aggravate OXPHOS dysfunction and affect mitophagy, producing a leakage of both mtDNA and proteins outside the mitochondria, which triggers an innate immune response, causing cardiovascular damage (Liu et al., 2022).

Mitochondria, the organelles responsible for generating energy for cellular metabolism (Cooper, 2000; Lodish et al., 2012; Chaban et al., 2014) contain several copies of their own genome, a circular double-stranded DNA molecule of ≈16.6 kb which in humans includes a total of 37 genes, 13 coding for the subunits of respiratory complexes I, III, IV, and V (Meiklejohn et al., 2013), 22 code for transfer RNAs (tRNAs) for the 20 standard amino acids, an extra gene for leucine and serine (Taanman, 1999; Cooper, 2000; Gray et al., 2008), and two for ribosomal RNAs (rRNAs) (Chan, 2006). The replication origin(s) and promoters for mtDNA are contained in an additional displacement loop (D-loop). Additionally, the cell nucleus contains genes encoding proteins related to mitochondria functions which regulate mtDNA transcription, replication, cell apoptosis and mitophagy, nucleotide biosynthesis, metabolism, and iron and calcium homeostasis (Timmis et al., 2004; Dolezal et al., 2006). Common maternally inherited mtDNA variants have been associated with CVD risk factors such as hypertension, diabetes, and dyslipidaemia (Calabrese et al., 2022). Recently, the role of mitochondrial genetic variants in the lipidomic context of CAD has been highlighted in 1,409 Han Chinese CAD patients, showing associations of D-loop variants with TG, Chol, LDL and HDL (Wang et al., 2021). Specific mitochondrial haplogroups have shown to confer a significant risk for many CAD related traits, such as coronary atherosclerosis (Sawabe et al., 2011), ischemic stroke (Tsai et al., 2020), myocardial infarction (Nishigaki et al., 2007b), atherosclerotic cerebral infarction (Nishigaki et al., 2007a), essential hypertension (Tsai et al., 2020) and T2DM in Asians (Fuku et al., 2007), and CVD (Veronese et al., 2019), atherosclerosis (Zhelankin et al., 2015; Piotrowska-Nowak et al., 2018), CAD (Kofler et al., 2009; Palacín et al., 2011), ischemic stroke (Rosa et al., 2008), hypertrophic cardiomyopathy (Castro et al., 2006; Singh et al., 2021) and diabetic retinopathy (Kofler et al., 2009; Estopinal et al., 2014; Bregman et al., 2017) in Europeans. Not all the studies however indicate an influence of mitochondrial variants in CAD related traits. No role for mtDNA variation was identified for hypertension or hyperglycaemia in participants from the Sympathetic activity and Ambulatory Blood Pressure in Africans (SABPA) prospective cohort study (Venter et al., 2017). A large study in over 9,000 Europeans failed to find a role for mitochondrial haplogroups on morbidity or mortality secondary to CVD (Benn et al., 2008). Therefore, the identification of mitochondrial genetic patterns and different forms of CVD and related traits is important to gain deeper understanding of the biological links between CVDs, lipid metabolism and clinical outcomes.

In this study, we aimed to investigate the role of genetic variants in both mtDNA and nuclear-encoded mitochondrial genes (NEMG) in cardiovascular diseases (CVD, CAD and hypertension) and cardiovascular risk factors (serum lipids: Chol, HDL, LDL, and TG) in a large population cohort (UK Biobank), additionally exploring the impact of diabetes.

## Methodology

### Ethics statement

This investigation conformed to the principles outlined in the Declaration of Helsinki. Participants gave informed consent prior to their inclusion in the UK Biobank project.

### Study design and population

**FIGURE 1:**
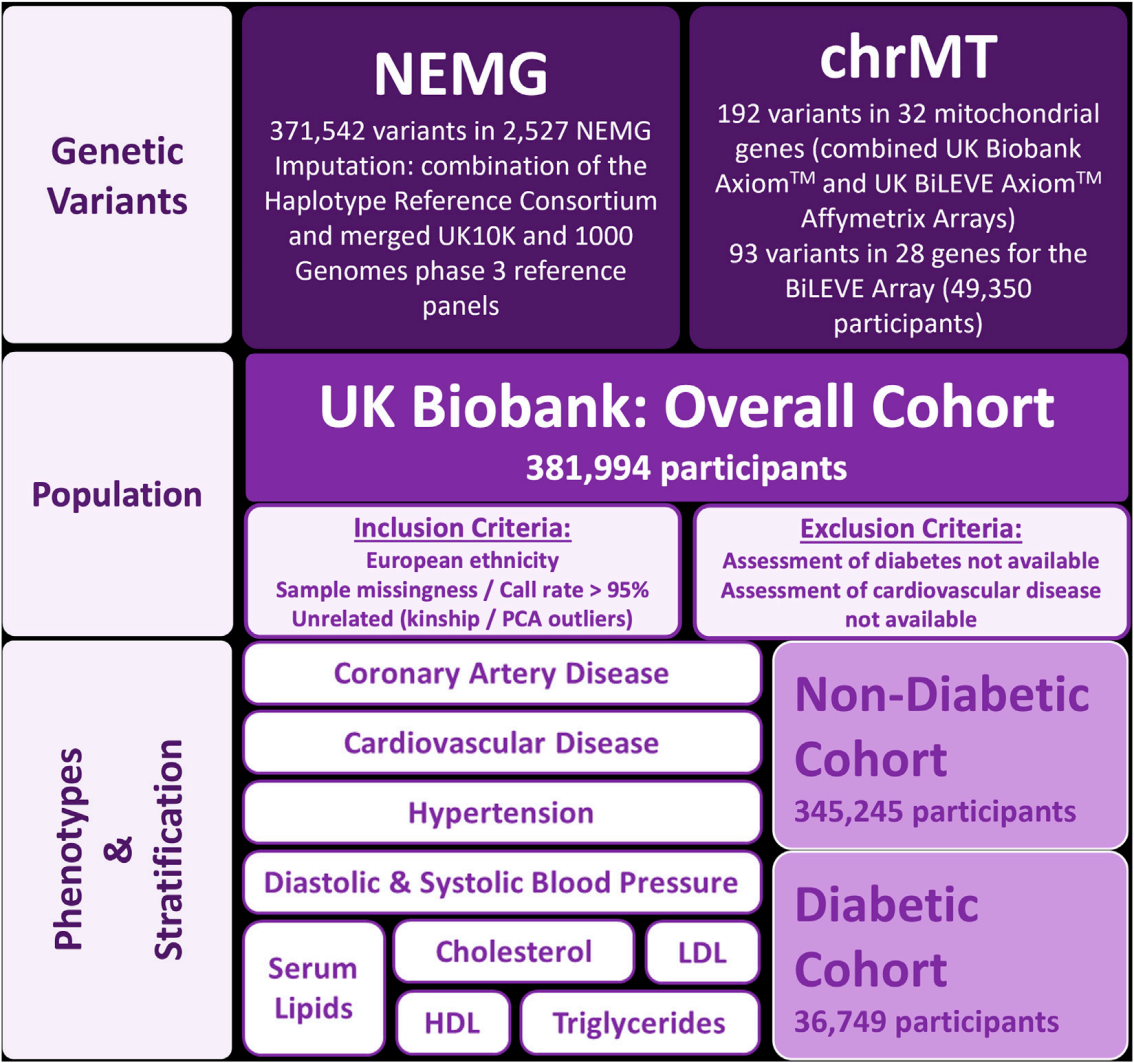
Design of the study. Abbreviations: chrMT: mitochondrial chromosome; HDL: high-density lipoprotein cholesterol; LDL: low-density lipoprotein cholesterol; NEMG: nuclear-encoded mitochondrial genes; PCA: principal component analysis.

This was a retrospective, cross-sectional study in participants of European ethnicity from the UK Biobank (Bycroft et al., 2018). To evaluate the effect of diabetes, the association of gene variants with the phenotypic outcomes were investigated with/without stratification by diabetes. Therefore, the total (overall) cohort was divided into two groups, according to the presence (diabetic cohort) or absence of diabetes (non-diabetic cohort) (Figure 1). Participants whose assessment of cardiovascular disease or diabetes was not possible were excluded from the analysis.

The UK Biobank project is a large-scale biomedical database and research resource providing genetic, lifestyle and health information from half a million UK participants (Bycroft et al., 2018).

### Phenotypic variables

#### Outcome variables

Outcome variables included CVD, CAD, hypertension, systolic and diastolic blood pressure (SBP, DBP) and serum lipids (cholesterol, HDL, LDL, and TG). Detailed definitions and disorders captured in every variable are provided within the “Supplementary Methods” section of the Supplementary Data Sheet.

### Genotyping and quality control

The Applied Biosystems™ UK Biobank Axiom™ and UK BiLEVE Axiom™ Affymetrix Arrays were used for genotyping by the UK Biobank. Genotypes were imputed by the UK Biobank using a combination of the Haplotype Reference Consortium and merged UK10K and 1000 Genomes phase 3 reference panels (Bycroft et al., 2018). PLINK 1.90 beta and PLINK 2.00 alpha were used to perform quality control (QC) and association analysis (Chang et al., 2015; Chang and GRAIL, 2020). Before QC, the study was comprised of 488,377 participants, 711,188 variants in NEMG and 265 mitochondrial variants. Individuals with high missingness rate or call rate lower than 95% were removed. Related individuals (identity by kinship coefficient >0.0884) and principal component analysis (PCA) outliers, as calculated by the UK Biobank, were also removed (Bycroft et al., 2018). Variants with minor allele frequency (MAF) < 1%, minor allele count (MAC) < 20 or variant call rate <95% were removed from the analysis. Autosomal variants not fulfilling Hardy-Weinberg equilibrium (HWE) (p < 1E-20) or imputation score under 0.3 were also excluded. After QC, 381,994 participants (Overall Cohort), 371,542 variants in 2,527 NEMG, along with 192 variants in 32 mitochondrial genes for the combined arrays and 93 variants in 28 genes for the BiLEVE array remained. For variants present in both arrays, only results in the UK Biobank Axiom™ were considered (largest sample).

### Mitochondrial haplogroups

Mitochondrial haplogroups were estimated using HaploGrep2 (Weissensteiner et al., 2016), based on PhyloTree17 (van Oven, 2015). Only the major European haplogroups H, V, HV, J, T, U, K, Z, W, X, I, and N were considered, grouping the remaining options in the “Other” category.

### Selection of nuclear-encoded mitochondrial genes

A total of 2,527 unique autosomal genes coding for 22,713 transcripts were investigated. The selection process produced 2,448 unique genes returned from database searches with a further 180 genes identified from literature searches for genes influencing mitochondrial function (Skelly, 2020). Briefly, several online databases and literature resources were searched for NEMGs: Mitoproteome (Taylor et al., 2003; Cotter et al., 2004; Pagliarini et al., 2008; Calvo et al., 2016), MitoMiner (Smith and Robinson, 2016), MitoMap (Brandon et al., 2005), Ensembl (Zerbino et al., 2018) and UniProt (The UniProt Consortium, 2017). Genes extracted from individual sources were reviewed and duplicates were excluded. Gene names were then screened to ensure there was no duplication between the database searches and literature searches. Genes were annotated with their official HUGO Gene Nomenclature Committee (HGNC) gene symbol (Wain et al., 2002) using Ensembl BioMart release 67 (May 2012) based on the February 2009 *Homo sapiens* high coverage assembly GRCh37 from the Genome Reference Consortium (Zerbino et al., 2018). Any genes not found in the BioMart (Zerbino et al., 2018) search were manually annotated according to their official HGNC gene symbol (Wain et al., 2002). The list of genes was then checked again for duplicates based on HGNC symbols, known pseudonyms and gene positions. Only genes found in autosomes were included in the analysis. Any genes on sex chromosomes, non-human genes, or bacterial artificial chromosomes were excluded from the final list of genes encoding proteins required for mitochondrial function.

### *In silico* analysis: functional annotation clustering

The online tool Functional Mapping and Annotation of Genome-wide Association Studies (FUMAGWAS) version 1.6.1 (Watanabe et al., 2017; 2019) was used to annotate, prioritise, visualise, and interpret the function of the genes statistically associated in the three cohorts. This tool automatically performs tissue specificity test and gene set/pathway enrichment analyses.

### Statistical analysis

#### Descriptive analysis

Descriptive and bivariate analyses were performed using R (R Core Team, 2024). Qualitative variables were expressed as percentage (%) of their total. Quantitative variables were expressed as the mean and the standard deviation.

#### Association analysis

Association analysis for individual variants was performed using PLINK 2.00 alpha using the “--glm” flag (Chang et al., 2015). For binary phenotypes (CVD, CAD and hypertension) --glm fits a logistic or Firth regression model (Chang et al., 2015). For quantitative phenotypes, --glm fits the linear model (Chang et al., 2015). Quantitative outcome variables were natural logarithmic transformed and analysed using the additional “--pheno-quantile-normalize” flag, to force quantitative phenotypes to a N (0, 1) distribution, preserving only the original rank orders (Chang et al., 2015). The --glm flag performs a multicollinearity check before each regression, which skips and reports “NA” results when it fails. Age, sex, genotyping batch and the 10 first PCAs values were included as covariates.

For the influence of traditional non-genomic risk factors for CVD on the outcomes (CAD, CVD and hypertension) multivariable logistic regression was performed in R (R Core Team, 2024).

#### Multiple comparisons correction

To correct for multiple testing, a Bonferroni correction for the number of independent variants (estimated using a pruning procedure of our data; r2 <0.2, window size 50 bp, offset 5 bp) after QC was used (Wuttke et al., 2016). The pruning estimated 47 independent variants for the mitochondrial chromosome for the combined arrays of the UK Biobank (35 when only the BiLEVE array was considered), yielding a threshold of 1E-03, and 57,457 variants for NEMG, yielding a threshold of 9E-07.

#### Clumping and annotation

Independent loci were identified using PLINK 1.90 beta clumping procedure (--clump-p1 5e-05 --clump-r2 0.1 --clump-kb 500) (Chang et al., 2015). A physical distance threshold for clumping of 1 kb was used for the mitochondrial chromosome. The independent loci were annotated using SNPnexus (Chelala et al., 2009; Dayem Ullah et al., 2012; 2013; Dayem Ullah et al., 2018; Oscanoa et al., 2020).

#### Mitochondrial haplogroups

Association analysis for mitochondrial haplogroups was performed using logistic regression in R version 4.3.0 (21/04/2023) (R Core Team, 2024), including as covariates age, sex and genotyping. Each haplogroup was analysed separately using all the other haplogroups as reference, after constructing dummy variables taking the values of 0 and 1, with the R package “fastDummies” (Kaplan, 2020). Principal components were not used as covariates to account for ancestry because of their potential correlation with haplogroups. The Bonferroni correction was applied to account for multiple comparisons, adjusting the *p*-value threshold, dividing by the number of haplogroups in each dataset (0.05/number of haplogroups).

#### Power calculations

Power calculations were performed for the CVD phenotype in the overall cohort and two strata using the Genetic Association Study (GAS) Power Calculator, considering a genotype relative risk of 1.2 (Skol et al., 2006), disease allele frequency of 0.02 and a prevalence of 32.2% (Einarson et al., 2018). In the cohort with diabetes, the statistical power was 84.2% and 94% for significance levels of 9E-07 and 1E-04, respectively; 100% for the other cohorts.

## Results

The descriptive analysis of the population is detailed in Table 1. Individuals with diabetes were more likely to be taking medication to control blood pressure or cholesterol, with more than half having CVD. Traditional non-genomic risk factors for CVD were associated with CAD, CVD and hypertension in the three cohorts (Supplementary Table S3).

**TABLE 1:** Descriptive analysis of the participants included in the study, stratified by diabetes. For qualitative variables, frequencies are expressed as number and percentage in brackets. Quantitative variables are expressed as mean and standard deviation.

	Overall cohort		Non-diabetic cohort		Diabetic cohort	
Variable	n	mean ± sd	n	mean ± sd	n	mean ± sd
Age (years)	381,994	57.1 ± 7.9	345,245	57.0 ± 8.0	36,749	58.8 ± 7.5
Body Mass Index (kg/m^2^)	380,779	27.5 ± 4.8	344,230	27.3 ± 4.6	36,549	29.5 ± 5.7
Diastolic blood pressure (mmHg)	381,683	82.4 ± 10.7	344,979	82.4 ± 10.7	36,704	81.7 ± 10.5
Systolic blood pressure (mmHg)	381,681	140.4 ± 19.7	344,977	140.2 ± 19.7	36,704	142.1 ± 19.2
Cholesterol (mmol/L)	364,321	5.7 ± 1.1	329,941	5.8 ± 1.1	34,380	5.1 ± 1.2
HDL cholesterol (mmol/L)	333,403	1.5 ± 0.4	301,859	1.5 ± 0.4	31,544	1.3 ± 0.4
LDL direct (mmol/L)	363,654	3.6 ± 0.9	329,351	3.6 ± 0.9	34,303	3.1 ± 0.9
Triglycerides (mmol/L)	364,027	1.8 ± 1.0	329,701	1.7 ± 1.0	34,326	2.0 ± 1.1

	n	n (%)	n	n (%)	n	n (%)
Sex (male)	381,994	173,246 (45.4)	345,245	153,454 (44.4)	36,749	19,792 (53.9)
Diabetes (yes)	381,994	36,749 (9.6)	345,245	0 (0.0)	36,749	36,749 (100.0)
Ever Smoker (yes)	380,621	174,716 (45.9)	344,059	155,909 (45.3)	36,562	18,807 (51.4)
Hypertensive Medication (yes)	381,388	95,356 (25.0)	344,788	77,692 (22.5)	36,600	17,664 (48.3)
Cholesterol Medication (yes)	381,436	72,626 (19.0)	344,821	54,325 (15.8)	36,615	18,301 (50.0)
Insulin (yes)	381,436	4,205 (1.1)	344,821	0 (0.0)	36,615	4,205 (11.5)
Cardiovascular Disease (yes)	381,994	134,950 (35.3)	345,245	114,127 (33.1)	36,749	20,823 (56.7)
Coronary Artery Disease (yes)	381,974	12,924 (3.4)	345,225	10,063 (2.9)	36,749	2,861 (7.8)
Hypertension (yes)	311,639	303,942 (97.5)	344,944	272,029 (78.9)	36,695	31,913 (87.0)

### Mitochondrial variants

**FIGURE 2:**
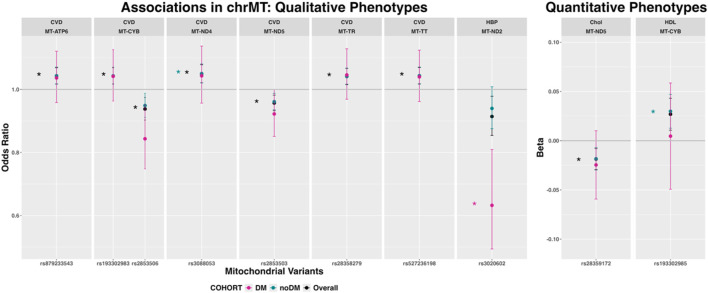
Mitochondrial variants associated with qualitative and quantitative phenotypes in any cohort (expressed as odds ratio and 95% confidence intervals for qualitative phenotypes and beta coefficients and 95% confidence intervals for quantitative phenotypes). Variants with p < 1E −03 are marked with an asterisk in the color of the corresponding cohort. The total number of participants per cohort was: 381,994 (overall cohort), 345,245 (non-diabetic cohort) and 36,749 (diabetic cohort). *p*-values correspond to the asymptotic *p*-value (or -log10(p)) for Z/chisq-stat (Qualitative variables, logistic regression) or for T/chisq-stat (Quantitative variables, linear regression). Abbreviations: chrMT: mitochondrial chromosome; Chol: total cholesterol; CVD: cardiovascular disease; DBP: diastolic blood pressure; DM: Diabetes Mellitus; HBP: hypertension; HDL: high-density lipoprotein cholesterol LDL: low-density lipoprotein cholesterol; SBP: systolic blood pressure.

Mitochondrial variants showed associations with CVD, hypertension, Chol and HDL (Figure 2). Full summary statistics are available in (Supplementary Document 1). Seven variants in *MT-ATP6*, *MT-CYB*, *MT-ND4*, MT*-ND5*, *MT-TR* and *MT-TT* were associated with CVD in the overall cohort (*MT-ND4*-rs3088053 also in the non-diabetic cohort). The *MT-ND2*-rs3020602 variant was associated with hypertension in the diabetic cohort. Directions of effects were consistent among cohorts.

### Mitochondrial haplogroups

**FIGURE 3:**
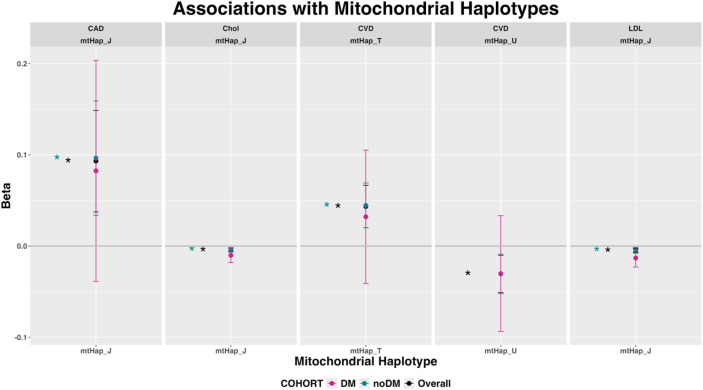
Mitochondrial haplogroups associated with phenotypes in any cohort. Associations with p < 4E-03 are marked with an asterisk in the color of the corresponding cohort (expressed as odds ratio and 95% confidence intervals for qualitative phenotypes and beta coefficients and 95% confidence intervals for quantitative phenotypes). The total number of participants per cohort was: 381,994 (overall cohort), 345,245 (non-diabetic cohort) and 36,749 (diabetic cohort). *p*-values correspond to the asymptotic *p*-value (or -log10(p)) for Z/chisq-stat (Qualitative variables, logistic regression) or for T/chisq-stat (Quantitative variables, linear regression). Abbreviations: chrMT: mitochondrial chromosome; Chol: total cholesterol; DBP: diastolic blood pressure; DM: Diabetes Mellitus; HBP: hypertension; HDL: high-density lipoprotein cholesterol LDL: low-density lipoprotein cholesterol; SBP: systolic blood pressure.

The frequency of the mitochondrial haplogroups in the UK Biobank Cohort is shown in Supplementary Table S4. Mitochondrial haplogroup J showed associations with CAD, Chol and LDL, whereas mitochondrial haplogroups T and U were associated with CVD. There were no significant associations in the diabetic cohort (Figure 3). Directions of effects were consistent among cohorts. The association of mitochondrial haplogroup T and CVD was consistent, showing associations with four of its defining mutations (*MT-ATP6*-rs879233543, *MT-TR*-rs28358279, *MT-CYB-*rs193302983 and *MT-TT*-rs527236198; Figure 2).

### NEMG variants

#### Significant associations across phenotypes

**FIGURE 4:**
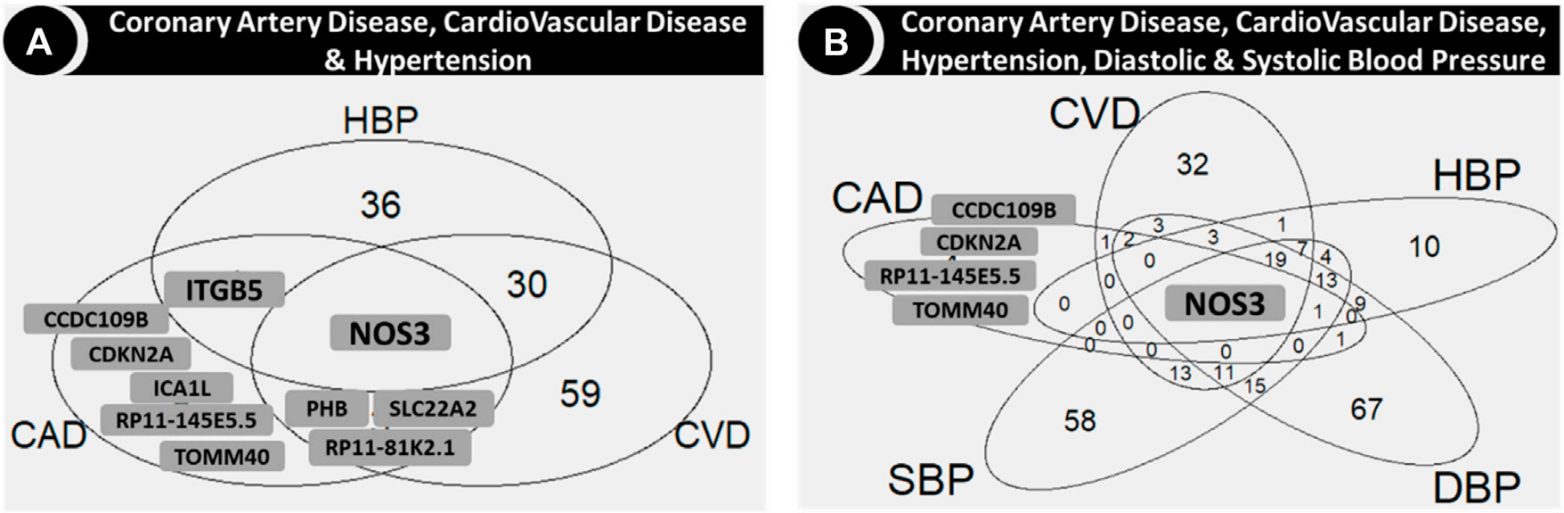
Venn diagrams showing the genes in the intersection among coronary artery disease, cardiovascular disease and hypertension **(A)** and also with diastolic and systolic blood pressure **(B)**. Abbreviations: CAD: coronary artery disease; CVD: cardiovascular disease; DBP: diastolic blood pressure; HBP: hypertension; SBP: systolic blood pressure.

**FIGURE 5:**
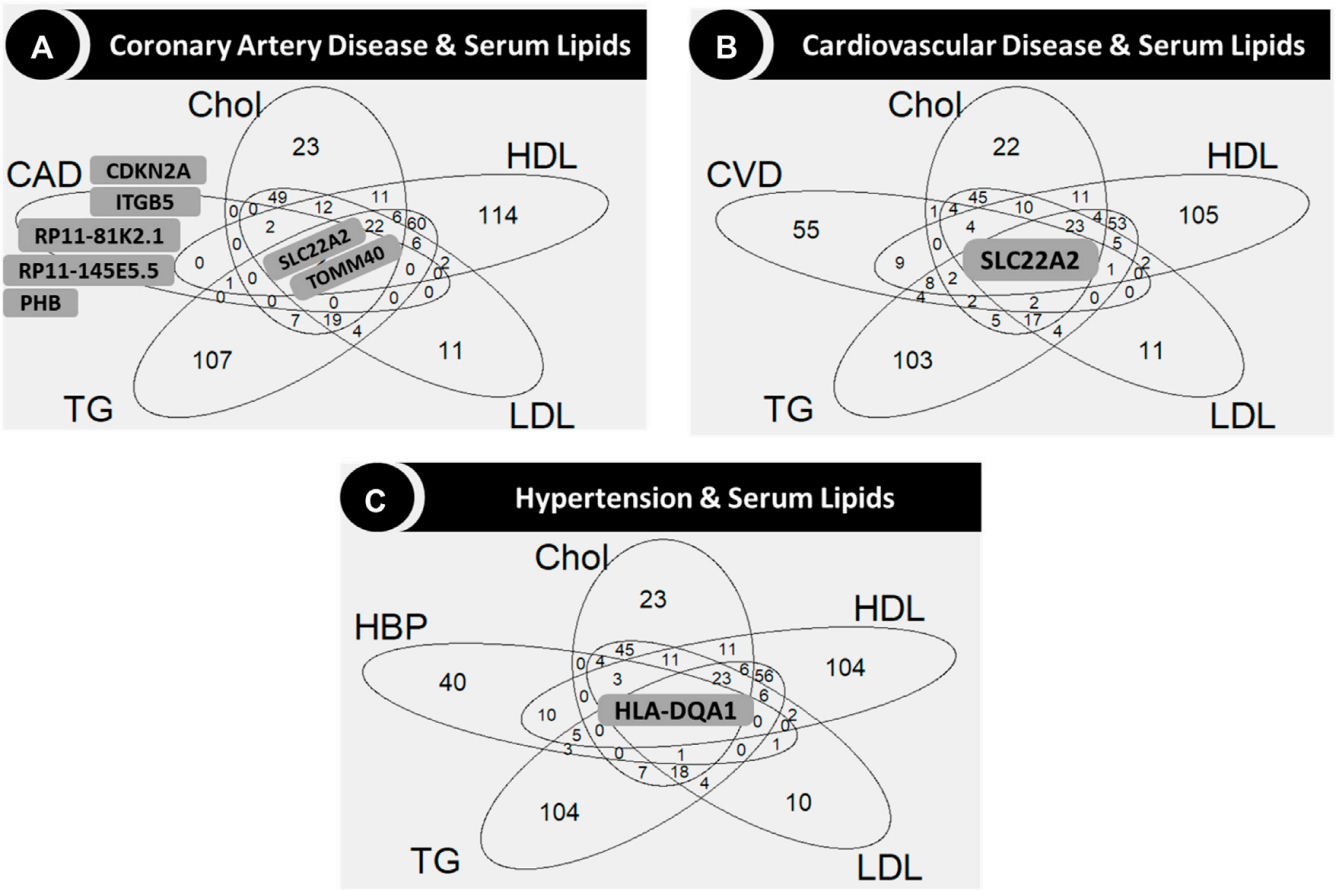
Venn diagrams showing the genes in the intersection among serum lipids and coronary artery disease **(A)**, cardiovascular disease **(B)** and hypertension **(C)**, respectively. Gene names are shown only for sets of five genes or less in the central (common to all phenotypes) and outer layers (uncommon genes). Abbreviations: CAD: coronary artery disease; Chol: total cholesterol; CVD: cardiovascular disease; DBP: diastolic blood pressure; HBP: hypertension; HDL: high-density lipoprotein cholesterol; LDL: low-density lipoprotein cholesterol; SBP: systolic blood pressure; TG: triglycerides.

Figures 4, 5 show the number of genes with associations to the different phenotypes, in any cohort. *NOS3* was common to CVD, CAD, hypertension, SBP and DBP (Figure 4). In particular, the *NOS3*-rs3918226T variant was associated with an increased risk of CVD, CAD, hypertension, and values of SBP and DBP and decreased serum levels of Chol and LDL, whereas the *NOS3*-rs891511A variant was associated with decreased SBP and DBP in the overall and/or non-diabetic cohorts (Supplementary Document 2). The *NOS3*-rs2070744 and *NOS3*-rs1007311 variants were also associated with HDL in the overall/non-diabetic cohorts.

*TOMM40*, one of the genes associated with CAD but not with CVD or the blood pressure related phenotypes (hypertension, SBP and DBP; Figure 4), was common for all the serum lipids, along with *SLC22A2* (Figure 5A), which was common for CVD and all serum lipids (Figure 5B). Two variants in *TOMM40* were associated with three phenotypes, rs34404554 (CAD, Chol and HDL) and rs61679753 (Chol, HDL and LDL) (Supplementary Document 2). Sixteen more variants were associated with Chol, HDL, LDL and TG (one or several) mainly in the overall and non-diabetic cohorts (Supplementary Document 2). As for *SLC22A2*, the rs10080815 variant was associated with CAD, CVD, Chol, LDL and TG; rs3127606 with CAD, Chol and LDL. Other 13 variants were associated with one or several phenotypes (CAD, CVD, Chol, HDL, LDL and TG), mainly in the overall and non-diabetic cohorts (Supplementary Document 2).

*HLA-DQA1* was associated with hypertension and all the serum lipids (Figure 5C). In particular, the rs6938008 variant was associated with hypertension and HDL, whereas the rs3129770 variant was associated with Chol, LDL and TG (Supplementary Document 2). Two variants were associated only in the cohort with diabetes (rs1048372 with HDL and rs9272417 with TG; Supplementary Document 2).

#### Significant associations in all cohorts

Sixty-six variants in 35 NEMG were consistently significant in all three cohorts for Chol, HDL, LDL, TG and/or DBP (Supplementary Figures S1–S5). Supplementary Table S5 shows the number of gene variants and genes associated with each phenotype, along with the number of traits reported in GWAS Catalog for those genes, according to FUMAGWAS (Watanabe et al., 2017). Among them, six variants in *TOMM40* were associated with LDL and Chol in all cohorts (rs71352238, rs2075650, rs1160983, rs11668327, rs111784051 and rs115881343). In addition, *TOMM40*-rs34404554 was associated with Chol and HDL in all cohorts (Supplementary Document 2). The GWAS Catalog reports *TOMM40* associations mainly with multiple serum lipid traits, including Chol, HDL, LDL and TG, C-reactive protein and body-mass index (BMI) (Supplementary Table S6).

Nine variants in *HLA-DQA1* were associated with LDL, TG and DBP in all cohorts (Supplementary Figures S3–S5). The *HLA-DQA1*-rs6938008 variant was also associated with hypertension in the overall cohort (Supplementary Document 2).

The rs7005363 variant in *MSRA* was associated with TG levels in all cohorts (along with other 12 in the overall/non-diabetic cohorts). Six other variants in this gene were also associated with CVD and hypertension in the overall/non-diabetic cohorts. In the enrichment analysis, *MSRA* appears along with *TOMM40* as cellular components of the mitochondrion and associated with serum metabolite levels, according to GWAS Catalog (Supplementary Table S6).

Other genes previously reported as risk factors for lipid traits and found significant in the three cohorts were *GCKR*, *SLC39A8*, *FADS2*, *PGS1*, *HNF4A* and *PLA2G6* (Supplementary Table S6).

#### Variants with different direction in the cohort with diabetes

**FIGURE 6:**
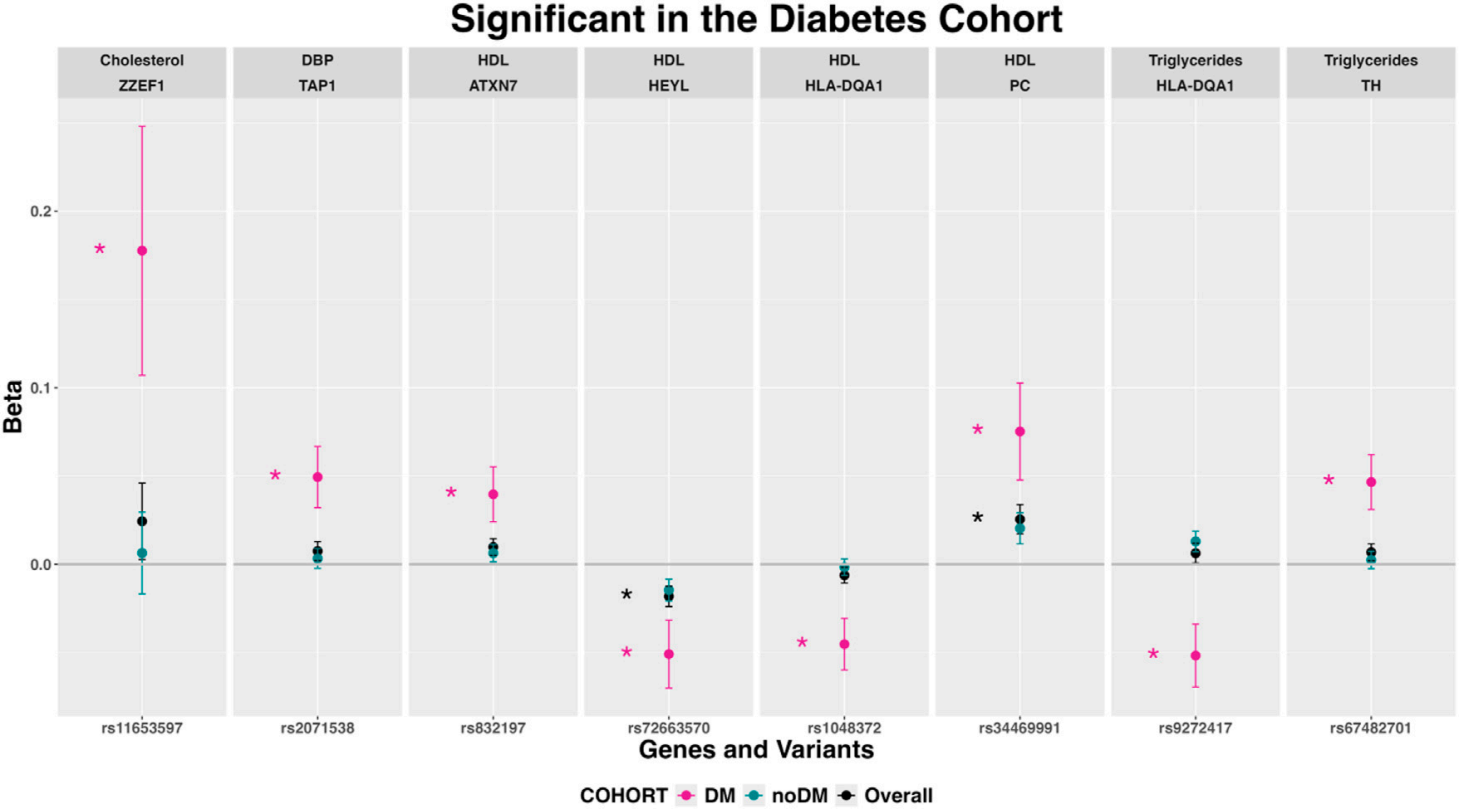
Variants in nuclear-encoded mitochondrial genes associated with several phenotypes after stratification by diabetes (expressed as beta coefficients and 95% confidence intervals). Abbreviations: chrMT: mitochondrial chromosome; Chol: total cholesterol; DM: Diabetes Mellitus; HBP: hypertension; HDL: high-density lipoprotein cholesterol.

Among the NEMG variants significantly associated in the overall and non-diabetic cohorts, some of them showed different direction of association in the diabetic cohort, although not significantly (Supplementary Figures S6–S12). As an exception to this, the *HLA-DQA1*-rs9272417 variant was significant only in the diabetic cohort (Figure 6; Supplementary Figure S10 and Supplementary Document 2).

#### Variants associated only in the cohort with diabetes

Eight variants in seven NEMG showed associations with HDL, TG, Chol and DBP only in participants with diabetes (Figure 6). In particular, two variants in HLA-DQA1 were associated with HDL (rs1048372) and TG (rs9272417).

## Discussion

Traditional non-genomic risk factors for CVD are associated with cardiac phenotypes in the UK Biobank cohort (Littlejohns et al., 2019; Razieh et al., 2022; Zhang et al., 2024). Although these clinical factors account for much of the CVD risk, it is valuable to explore the association between genomic factors, including mitochondrial DNA variation and variation in mitochondrial related genes, and cardiac phenotypes.

### mtDNA

Our study shows a consistent association between mitochondrial haplogroup T and CVD, reflected through associations not only with the haplogroup itself, but also with four of its defining mutations. The importance of this mitochondrial haplogroup on CVD had been previously evidenced by more than 3.5 times increase in the risk of hypertrophic cardiomyopathy in males, the most common genetic disorder of the heart (Castro et al., 2006; Singh et al., 2021). The mtDNA haplogroup T was associated with higher risk of CAD (14.8% vs. 8.3%; *p* = 0.002) in the study of Middle European Caucasians, including 487 patients with angiographically documented CAD and 1,527 control subjects without clinical manifestations of atherosclerotic disease (Kofler et al., 2009). One of the defining variants of haplotype T (*MT-TT-*rs527236198A) has recently been associated with higher risk of CAD in Iranian patients, demonstrating a transethnic effect (Heidari et al., 2020). Furthermore, JT haplogroups (HR = 0.75; 95%CI: 0.54–0.96; *p* = 0.03), and particularly J (HR = 0.71; 95%CI: 0.46–0.95; *p* = 0.02) have been associated with a reduced risk of CVD after a median follow-up of 8 years in 3,288 Caucasian participants (Veronese et al., 2019). In our study, mitochondrial haplogroup J was associated with higher risk of CVD along with lower levels of Chol and LDL. Interestingly, *MT-ND5*-rs28359172, one of the defining mutations of mitochondrial haplogroup J, was also associated with Chol levels in 321,188 individuals. We have recently observed this variant associated with eGFR levels in 329,235 participants from the UK Biobank (Cañadas-Garre et al., 2024). Other variants in *MT-ND5* (rs2853503), and *MT-CYB*-rs2853506, both associated with CVD in our analysis, were also associated with renal function in previous works in the UK Biobank (SCr, SCysC and eGFR) (Yonova-Doing et al., 2021; Cañadas-Garre et al., 2024) *MT-ND5* encodes the NADH dehydrogenase 5 subunit gene. Mutations in *MT-ND5* have been associated with tubulo-interstitial kidney disease, clinically characterised by proteinuria and hypertension (Bakis et al., 2020), which could partially explain its role in the overlap between CKD and CVD. Mitochondrial genes like *MT-ATP6*, *MT-CYB*, *MT-ND4*, *MT-ND5*, *MT-TR* and *MT-TT*, all associated with CVD in our study, have also been associated with CVD in other ethnic populations, e.g., CAD in Iranians (Heidari et al., 2020), and hypertension and ischaemic stroke in Chinese (Zhu et al., 2016; 2018; Guo et al., 2022). Mitochondrial genes, particularly those coding the oxidative phosphorylation (OXPHOS) enzyme complexes I-IV, play significant roles in CVD due to their involvement in mitochondrial function and energy production. Variants in these genes can disrupt normal electron transport chain activity, leading to decreased ATP levels and increased oxidative stress, causing mitochondrial dysfunction, which is increasingly recognized as a contributing factor to various forms of heart disease, including CAD and cardiomyopathies (Venter et al., 2018; Campbell et al., 2022). Many of the conditions causing CVD, such as atherosclerosis, hypertension, cardiomyopathy and T2DM, are associated with inflammation caused by oxidative stress (Venter et al., 2018).

### NEMG

#### NOS3

The findings of our study place *NOS3* as the gene most consistently associated with CVD, CAD and blood pressure related traits (hypertension, SBP and DBP). *NOS3*, encoding for nitric oxide synthase 3, may play a role in the development and progression of CAD (Nikpay et al., 2015; Van Der Harst and Verweij, 2018; Zhou et al., 2018; Hartiala et al., 2021; Aragam et al., 2022; Temprano-Sagrera et al., 2022; Rai et al., 2023), which appears to be mediated mainly through blood pressure regulation across ancestries, according to many GWAS (Liu et al., 2016; Hoffmann et al., 2017; Wain et al., 2017; Feitosa et al., 2018; Sung et al., 2018; Kichaev et al., 2019; Giri et al., 2019; Wojcik et al., 2019; Sakaue et al., 2021; Plotnikov et al., 2022; Schoeler et al., 2023). Our results confirm the role of *NOS3*-rs3918226T as a marker of susceptibility for CAD (Nikpay et al., 2015; Van Der Harst and Verweij, 2018; Zhou et al., 2018; Aragam et al., 2022; Temprano-Sagrera et al., 2022) and CVD (Kichaev et al., 2019), plus *NOS3*-rs891511A as a potential reducer of blood pressure (Liu et al., 2016; Hoffmann et al., 2017; Wain et al., 2017; Feitosa et al., 2018; Sung et al., 2018; Kichaev et al., 2019; Giri et al., 2019; Wojcik et al., 2019; Sakaue et al., 2021; Plotnikov et al., 2022; Schoeler et al., 2023). We have recently reported the association of these two variants in *NOS3* (rs3918226 and rs891511) with kidney damage in the UK Biobank cohort (Cañadas-Garre et al., 2024). Other variants in *NOS3* have shown a clear link with end-stage renal disease (ESRD) (Elsaid et al., 2021; Padhi et al., 2022), chronic kidney disease (CKD) (Gunawan et al., 2020), CKD progression (Medina et al., 2018) and diabetic kidney disease (DKD) (Chen et al., 2016; Roumeliotis et al., 2021). For patients with ESRD receiving haemodialysis, CVD is the major cause of morbidity and mortality (Fox et al., 2012; Mahmoodi et al., 2012) and CVD is present in over 50% of them (Cozzolino et al., 2018). Longitudinal cohorts such as the UK Biobank in time will allow further investigation of common genetic risk factors contributing to early detection, predisposition and multimorbidity for both CVD and ESRD.

#### SLC22A2

In our study, up to 15 variants in *SLC22A2* gene were associated with CAD and CVD and all serum lipids, but none of the blood pressure related phenotypes. *SLC22A2* encodes the solute carrier family 22 member 2, a polyspecific organic cation transporter responsible from elimination of endogenous small organic cations, toxins and drugs (Gründemann and Schömig, 2000). These *SLC22A2* gene variant associations confirm results from many previous GWAS identifying *SLC22A2* not only as a susceptibility risk factor for CAD (Nikpay et al., 2015; Yeo et al., 2017; Lempiäinen et al., 2018; Van Der Harst and Verweij, 2018; Svishcheva et al., 2019; Shadrina et al., 2020; Aragam et al., 2022), but also as a marker of lipoprotein (a) levels (Mack et al., 2017; Liu et al., 2019; Sinnott-Armstrong et al., 2021), a well-known genetically determined risk factor of CAD (Berg et al., 1979; Tipping et al., 2009; Schatz et al., 2017; Foscolou et al., 2018). Regarding the association of *SLC22A2* with serum lipid levels, as in our study, many others have found variants in the *SLC22A2* gene influencing serum levels of atherogenic risk lipids, and potentially impacting lipid metabolism (Bar et al., 2020; Lotta et al., 2021; Sakaue et al., 2021; Sinnott-Armstrong et al., 2021; Koskeridis et al., 2022; Richardson et al., 2022; Davyson et al., 2023; Schoeler et al., 2023). Altogether, these findings indicate that *SLC22A2* may play a role in regulating serum lipid levels, thereby potentially influencing the risk of atherosclerosis and CAD. *SLC22A2* has been implicated in the regulation of plasma lactate levels, particularly in the context of CVD and T2DM, with TT-carriers of the *SLCA22A2*-rs316019 variant showing significantly higher fasting plasma lactate concentrations (Li et al., 2010). Increased lactatemia has shown to be a marker of poor prognosis in patients with acute heart failure (Ouyang et al., 2023). The influence of *SLC22A2* variants on lactatemia could not be assessed, as our UK Biobank application did not include the participants metabolomics profiling, where lactate levels were measured. The relevance of *SLC22A2* goes beyond CAD, since it is a shared susceptibility locus for T2DM, with common etiological pathways between them (Zhao et al., 2017; Xue et al., 2018; Ray and Chatterjee, 2020). Furthermore, we and others have previously demonstrated the importance of *SLC22A2* in CKD, renal traits and function (Chambers et al., 2010; Köttgen et al., 2010; Pattaro et al., 2016; Cañadas-Garre et al., 2024), thus revealing one of the many potential biological connections between the etiologies of CVD and CKD. Of interest, CKD is one of the most important risk factors for the development of CVD, and most patients with CKD die from cardiovascular causes before they progress to kidney failure (Liu et al., 2014; Jankowski et al., 2021; Warrens et al., 2022; Zoccali et al., 2023). In fact, a recent study has highlighted the common genetic architectures overlapped between CAD and CKD using summary statistics publicly available from large scale GWAS, showing *NOS3*, *SLC22A2* and *TOMM40* among the genes with potential pleiotropy between these two conditions (Chen et al., 2020).

#### TOMM40

Among the NEMG investigated in our study, *TOMM40* was associated with CAD and all the serum lipids, but not with CVD or blood pressure related traits. These results reinforce the robust association between the G-allele of *TOMM40*-rs2075650 and increased risk of CAD identified in GWAS (Middelberg et al., 2011; Deloukas et al., 2013; Christiansen et al., 2017b; Feng et al., 2017). *TOMM40* codes for the channel-forming subunit of the translocase of the mitochondrial outer membrane (TOM) complex 40, essential for protein import into mitochondria (Humphries et al., 2005). The most investigated variant is rs2075650, located in an intronic region of the *TOMM40* gene, just upstream of *APOE*, and *APOC1*, holding a relatively strong linkage disequilibrium that has suggested the potential causal variation to relay on the *APOE* gene (Deelen et al., 2011; Christiansen et al., 2017a; Palmer et al., 2021). Variants in *TOMM40* have also been proposed as predictors of non-HDL-Chol in 2,800 African-Americans (Feng et al., 2017), LDL (Sandhu et al., 2008; Talmud et al., 2009; Middelberg et al., 2011; Radovica et al., 2014) and TG (Salakhov et al., 2014) in Europeans and dyslipidaemia in 1,962 Chinese Maonans (Miao et al., 2018). In our study, the rs2075650 variant was consistently associated with LDL and Chol serum levels in all cohorts, with the G allele reducing HDL levels and increasing the rest of the serum lipids and the risk of CAD, evidencing again the crucial role of this gene in the biological pathways involving serum lipids.

In addition to CAD and dyslipidemia, genetic variants in *TOMM40* have been investigated in other contexts, having been associated with reduced BMI (Guo et al., 2013), lower levels of high-sensitivity C-reactive protein (hs-CRP) (Ellis et al., 2014; Christiansen et al., 2017a), healthy aging and longevity (Deelen et al., 2011; 2014; Chen et al., 2022; Torres et al., 2022) and increased risk of Alzheimer’s disease (Denny et al., 2013; Davies et al., 2015). A recent systematic review identified the rs2075650 and rs10524523 variants as the two most commonly associated with longevity. The outcomes associated with *TOMM40* variants were changes in BMI, brain integrity, cognitive functions, altered inflammatory network, vulnerability to vascular risk factors (including hypertension, hyperlipidemia, and diabetes, among others), and longevity (Gui et al., 2021; Chen et al., 2022). Interestingly, *TOMM40* polymorphisms strongly interact with vascular risk factors to influence cognitive performance, being markedly detrimental to cognition (Gui et al., 2021). Further analyses revealed *TOMM40*-rs2075650G allele also interacted with diabetes, dramatically reducing the Mini-Mental State Examination score, used to evaluate cognitive impairment (Gui et al., 2021). In line with this, other *TOMM40* variant previously linked to Alzheimer’s disease (Nazarian et al., 2019) and cerebral amyloid deposition (Yan et al., 2021) is rs71352238, found consistently associated with LDL and Chol levels in all cohorts in our study, bringing more evidence to the link between *TOMM40*, serum lipids and development of Alzheimer’s disease.

#### HLA-DQA1

Our study identified a consistent association for *HLA-DQA1* with hypertension and serum lipids, with up to nine variants associated with LDL, TG and DBP in all cohorts. The *HLA-DQA1* gene, also known as Major Histocompatibility Complex, Class II, DQ Alpha 1, is part of the human leukocyte antigen (HLA) complex, which plays a critical role in the immune system by presenting antigens to CD4-positive T-lymphocytes. Variants of the *HLA-DQA1* gene have been associated with various autoimmune conditions, including T1DM (Scott et al., 2017; Onengut-Gumuscu et al., 2019; Liao et al., 2023), and have been proposed as markers of susceptibility for T2DM and diabetic nephropathy (Ma et al., 2013). The HLA-DQA1*0501 allele confers susceptibility to idiopathic dilated cardiomyopathy, while the *DQA1* 0201 allele provides protection (Limas et al., 1995; Liu et al., 2005). Unfortunately, the tag SNPs for these two markers were not among our postQC variants. In the context of hypertension, they have been proposed as a novel genetic risk and prognostic factor for pulmonary arterial hypertension in systemic lupus erythematosus patients (Qian et al., 2023), are associated with heart disease, stroke, diabetes, and hypertension among subjects with Graves’ disease (Liao et al., 2022) and may influence hypertension and renal outcomes in patients with membranous nephropathy (Fan et al., 2021). However, a direct association of *HLA-DQA1* with serum lipids is not reported in the literature. Our study suggests a potential role for *HLA-DQA1* in biological context for the development of hypertension in patients with altered serum lipid levels.

### Variants associated only in the cohort with diabetes

Among the associations only found in participants with diabetes, a *TAP1* gene variant was associated with DBP. The transport associated with antigen processing 1 (*TAP1*) gene polymorphism at codon 637 is associated with hypertension with the GG genotype being linked to higher SBP and DBP (Shen et al., 2007). However, the exact biological role of *TAP1* in the pathophysiology of hypertension is not yet fully understood.

We found the *HEYL* gene, encoding a member of the hairy and enhancer of split-related (HESR) family of basic helix-loop-helix (bHLH)-type transcription factors, associated with HDL only in the diabetic cohort. Although we have not found a specific relationship between the *HEYL* gene and HDL levels in the literature, HDL protects against inflammatory activation of the arterial system and may be involved in the regulation of Notch signaling, which in turn can impact the expression of *HEYL* and other related genes (Briot et al., 2016). In our participants with diabetes, the *HLA-DQA1*-rs1048372 variant showed a significant and different direction of effect over serum TG levels, compared with participants without diabetes. A recent GWAS in 56,664 individuals has identified other variant in *HLA-DQA1* (rs17426593) associated with hypothyroidism (Kim and Park, 2023). In those individuals, the serum TG concentrations were also positively associated with hypothyroidism risk (Kim and Park, 2023). But given that no specific relationship between either *HEYL* and HDL levels or *HLA-DQA1* and TG levels has been reported yet, further research may be needed to fully understand the connection between these genes and serum lipid levels.

A further variant with significant association with TG in participants with diabetes mapped to the tyrosine hydroxylase (*TH*) gene, coding an enzyme that catalyzes the first step in the synthesis of catecholamines, such as dopamine and noradrenaline. Reduced *TH* expression in brown adipose tissue can impact various physiological processes, including lipid metabolism. In *TH* heterozygous mice, the reduction of TH in brown adipose tissue affected the catecholaminergic response to cold exposure, leading to implications for cold adaptation (Vázquez et al., 2018). In rats, knockdown in the hypothalamus led to elevated plasma TG levels, inducing obesity and glucose intolerance (Zhang et al., 2023). Furthermore, there is evidence that circulating TG can influence dopamine-associated behaviours (Berland et al., 2020). These findings suggest a potential link between TH and triglycerides, indicating a potential role in lipid metabolism and related physiological functions.

For the rest of the genes with associations only in the cohort with diabetes (*ZZEF1* and Chol, *ATXN7*, *PC*, *HLA-DQA1* and HDL), this study is the first to identify an association and more research will be required to establish a comprehensive understanding of their impact.

## Limitations

We used a relatively large cohort, the UK Biobank, to investigate an extensive selection of variants in both mtDNA and NEMG in this cross-sectional study with sufficient power to detect associations in common variants, but our power was reduced for less common variants in sub-group analysis for diabetes (84.2% for NEMG, 94% for mitochondrial variants). Nonetheless, we have confirmed known and identified novel significant associations with CVDs. We have explored a variety of traits and approached cardiovascular conditions through three different phenotypes (CVD *per se*, CAD and hypertension) using standard definitions taken from the disease and medication information provided by the UK Biobank, combining data from different variables; these definitions, based on variables participant operations (Data Field #20004), non-cancer illness (Data Field #20002), and other medications (Data Field #20003) as ICD-10 codes were not available, may differ from those used by other authors. Although our study was limited to participants with European ancestry, it successfully identified mitochondrial gene variation associated with cardiovascular traits that have also been reported in other ethnic populations; however broader investigation in appropriately powered cohorts with all ethnicities would be necessary to confirm associations in diverse groups. Our study has also pinpointed mitochondrial and NEMGs variants capable to influence multiple phenotypes, as common nexus between CAD, CVD and hypertension, exhibiting pleiotropy or as a consequence of a shared genetic structure in these conditions. However, determining whether a phenotype is specifically associated with mitochondrial related variants, or influenced by other factors may be quite complex due to the inherent variability and pleiotropy of mtDNA variants.

## Conclusion

Our study highlights the relevance of variants in both mitochondrial genes and NEMG in the context of CVDs, especially CAD and hypertension, and CVD modifiable risk factors such as serum lipids in people with and without diabetes. We have linked mitochondrial haplogroup U to CVD and consistently demonstrated an association between mitochondrial haplogroups J and T and CVD, confirming previous results. We have also proposed new markers of hypertension and serum lipids in the context of diabetes. The findings of our study also make evident connections between the etiological pathways underlying CVDs, blood pressure and serum lipids. Our results place *NOS3* gene as the common nexus between CAD, CVD and hypertension, with serum lipids connected to CVD through *SLC22A2*, in combination with *TOMM40* for CAD and to hypertension through *HLA-DQA1*.

These results may help future endeavors examining the common mechanisms underlying these traits to elucidate the biological pathways responsible for CVDs.

## Data Availability

The original contributions presented in the study are included in the article/Supplementary Material, further inquiries can be directed to the corresponding author. The data analyzed in this study was obtained from the UK Biobank, through application number 14259.

## Abbreviations

ACEi, angiotensin-converting enzyme (ACE) inhibitors; ARBs, angiotensin II receptor blockers; CABG, coronary artery bypass graft; CAD, coronary artery disease; Chol, cholesterol; CKD, chronic kidney disease; CVD, cardiovascular disease; DBP, diastolic blood pressure; DKD, diabetic kidney disease; DM, diabetes mellitus; ESRD, end-stage renal disease; HDL, high-density lipoproteins; LDL, low-density lipoproteins; NEMG, nuclear-encoded mitochondrial genes; PTCA, percutaneous transluminal coronary angioplasty; RAASi, renin-angiotensin-aldosterone system inhibitors; SBP, systolic blood pressure; SNP, single nucleotide polymorphism; TG, triglycerides.

## References

[B1] Aragam K. G. Jiang T. Goel A. Kanoni S. Wolford B. N. Atri D. S. (2022). Discovery and systematic characterization of risk variants and genes for coronary artery disease in over a million participants. Nat. Genet. 54, 1803–1815. 10.1038/s41588-022-01233-6 36474045 PMC9729111

[B2] Bakis H. Trimouille A. Vermorel A. Redonnet I. Goizet C. Boulestreau R. (2020). Adult onset tubulo-interstitial nephropathy in MT-ND5-related phenotypes. Clin. Genet. 97, 628–633. 10.1111/CGE.13670 31713837

[B3] Bar N. Korem T. Weissbrod O. Zeevi D. Rothschild D. Leviatan S. (2020). A reference map of potential determinants for the human serum metabolome. Nature 588, 135–140. 10.1038/S41586-020-2896-2 33177712

[B4] Benn M. Schwartz M. Nordestgaard B. G. Tybjaerg-Hansen A. (2008). Mitochondrial haplogroups: ischemic cardiovascular disease, other diseases, mortality, and longevity in the general population. Circulation 117, 2492–2501. 10.1161/CIRCULATIONAHA.107.756809 18458168

[B5] Berg K. Dahlen G. Borresen A. L. (1979). Lp(a) phenotypes, other lipoprotein parameters, and a family history of coronary heart disease in middle-aged males. Clin. Genet. 16, 347–352. 10.1111/J.1399-0004.1979.TB01014.X 230000

[B6] Berland C. Montalban E. Perrin E. Di Miceli M. Nakamura Y. Martinat M. (2020). Circulating triglycerides gate dopamine-associated behaviors through DRD2-expressing neurons. Cell Metab. 31, 773–790. 10.1016/J.CMET.2020.02.010 32142669 PMC7250662

[B7] Brandon M. C. Lott M. T. Nguyen K. C. Spolim S. Navathe S. B. Baldi P. (2005). MITOMAP: a human mitochondrial genome database—2004 update. Nucleic Acids Res. 33, D611–D613. 10.1093/nar/gki079 15608272 PMC540033

[B8] Bregman J. A. Herren D. J. Estopinal C. B. Chocron I. M. Harlow P. A. Warden C. (2017). Mitochondrial haplogroups affect severity but not prevalence of diabetic retinopathy. Invest Ophthalmol. Vis. Sci. 58, 1346–1351. 10.1167/iovs.16-20616 28245487 PMC5341621

[B9] Briot A. Bouloumié A. Iruela-Arispe M. L. (2016). Notch, lipids, and endothelial cells. Curr. Opin. Lipidol. 27, 513–520. 10.1097/MOL.0000000000000337 27454451 PMC5340259

[B10] Bycroft C. Freeman C. Petkova D. Band G. Elliott L. T. Sharp K. (2018). The UK Biobank resource with deep phenotyping and genomic data. Nature 562, 203–209. 10.1038/s41586-018-0579-z 30305743 PMC6786975

[B11] Cadby G. Melton P. E. McCarthy N. S. Giles C. Mellett N. A. Huynh K. (2020). Heritability of 596 lipid species and genetic correlation with cardiovascular traits in the Busselton Family Heart Study. J. Lipid Res. 61, 537–545. 10.1194/jlr.RA119000594 32060071 PMC7112151

[B12] Calabrese C. Pyle A. Griffin H. Coxhead J. Hussain R. Braund P. S. (2022). Heteroplasmic mitochondrial DNA variants in cardiovascular diseases. PLoS Genet. 18, e1010068. 10.1371/JOURNAL.PGEN.1010068 35363781 PMC9007378

[B13] Calvo S. E. Clauser K. R. Mootha V. K. (2016). MitoCarta2.0: an updated inventory of mammalian mitochondrial proteins. Nucleic Acids Res. 44, D1251–D1257. 10.1093/nar/gkv1003 26450961 PMC4702768

[B14] Campbell T. Slone J. Huang T. (2022). Mitochondrial genome variants as a cause of mitochondrial cardiomyopathy. Cells 11, 2835. 10.3390/CELLS11182835 36139411 PMC9496904

[B15] Cañadas-Garre M. Baños-Jaime B. Maqueda J. J. Smyth L. J. Cappa R. Skelly R. (2024). Genetic variants affecting mitochondrial function provide further insights for kidney disease. BMC Genomics 25, 576. 10.1186/S12864-024-10449-1 38858654 PMC11163707

[B16] Cañadas-Garre M. Kunzmann A. T. Anderson K. Brennan E. P. Doyle R. Patterson C. C. (2023). Albuminuria-related genetic biomarkers: replication and predictive evaluation in individuals with and without diabetes from the UK Biobank. Int. J. Mol. Sci. 24, 11209. 10.3390/ijms241311209 37446387 PMC10342310

[B17] Castro M. G. Huerta C. Reguero J. R. Soto M. I. Domenech E. Alvarez V. (2006). Mitochondrial DNA haplogroups in Spanish patients with hypertrophic cardiomyopathy. Int. J. Cardiol. 112, 202–206. 10.1016/j.ijcard.2005.09.008 16313983

[B18] CDC (2023). Coronary artery disease | cdc.gov. Available at: https://www.cdc.gov/heartdisease/coronary_ad.htm (Accessed December 28, 2023).

[B19] Chaban Y. Boekema E. J. Dudkina N. V. (2014). Structures of mitochondrial oxidative phosphorylation supercomplexes and mechanisms for their stabilisation. Biochim. Biophys. Acta 1837, 418–426. 10.1016/j.bbabio.2013.10.004 24183696

[B20] Chambers J. C. Zhang W. Lord G. M. van der Harst P. Lawlor D. A. Sehmi J. S. (2010). Genetic loci influencing kidney function and chronic kidney disease. Nat. Genet. 42, 373–375. 10.1038/ng.566 20383145 PMC3748585

[B21] Chan D. C. (2006). Mitochondria: dynamic organelles in disease, aging, and development. Cell 125, 1241–1252. 10.1016/j.cell.2006.06.010 16814712

[B22] Chang C. Grail I. (2020). Human longevity, I., and department of biomedical data science, S PLINK 2.00 alpha. Available at: https://www.cog-genomics.org/plink/2.0/.

[B23] Chang C. C. Chow C. C. Tellier L. C. A. M. Vattikuti S. Purcell S. M. Lee J. J. (2015). Second-generation PLINK: rising to the challenge of larger and richer datasets. Gigascience 4, 7–16. 10.1186/s13742-015-0047-8 25722852 PMC4342193

[B24] Chelala C. Khan A. Lemoine N. R. (2009). SNPnexus: a web database for functional annotation of newly discovered and public domain single nucleotide polymorphisms. Bioinformatics 25, 655–661. 10.1093/bioinformatics/btn653 19098027 PMC2647830

[B25] Chen F. Li Y.-M. Yang L.-Q. Zhong C.-G. Zhuang Z.-X. (2016). Association of NOS2 and NOS3 gene polymorphisms with susceptibility to type 2 diabetes mellitus and diabetic nephropathy in the Chinese Han population. IUBMB Life 68, 516–525. 10.1002/iub.1513 27192959

[B26] Chen H. Wang T. Yang J. Huang S. Zeng P. (2020). Improved detection of potentially pleiotropic genes in coronary artery disease and chronic kidney disease using GWAS summary statistics. Front. Genet. 11, 592461. 10.3389/FGENE.2020.592461 33343632 PMC7744760

[B27] Chen S. Sarasua S. M. Davis N. J. DeLuca J. M. Boccuto L. Thielke S. M. (2022). TOMM40 genetic variants associated with healthy aging and longevity: a systematic review. BMC Geriatr. 22, 667. 10.1186/s12877-022-03337-4 35964003 PMC9375314

[B28] Christiansen M. K. Larsen S. B. Nyegaard M. Neergaard-Petersen S. Ajjan R. Würtz M. (2017a). Coronary artery disease-associated genetic variants and biomarkers of inflammation. PLoS One 12, e0180365. 10.1371/journal.pone.0180365 28686695 PMC5501546

[B29] Christiansen M. K. Nyegaard M. Larsen S. B. Grove E. L. Würtz M. Neergaard-Petersen S. (2017b). A genetic risk score predicts cardiovascular events in patients with stable coronary artery disease. Int. J. Cardiol. 241, 411–416. 10.1016/j.ijcard.2017.04.045 28442232

[B30] Companioni O. Rodríguez Esparragón F. Fernández-Aceituno A. M. Rodríguez Pérez J. C. (2011). Genetic variants, cardiovascular risk and genome-wide association studies. Rev. Esp. Cardiol. 64, 509–514. 10.1016/j.recesp.2011.01.010 21550161

[B31] Cooper G. (2000). The cell: a molecular approach. 2nd Edn. Sunderland (MA): Sinauer Associates.

[B32] Coronary Artery Disease (CAD) (2023). Symptoms and treatment. Available at: https://my.clevelandclinic.org/health/diseases/16898-coronary-artery-disease (Accessed December 28, 2023).

[B33] Cotter D. Guda P. Fahy E. Subramaniam S. (2004). MitoProteome: mitochondrial protein sequence database and annotation system. Nucleic Acids Res. 32, D463–D467. 10.1093/nar/gkh048 14681458 PMC308782

[B34] Cozzolino M. Mangano M. Stucchi A. Ciceri P. Conte F. Galassi A. (2018). Cardiovascular disease in dialysis patients. Nephrol. Dial. Transplant. 33, iii28–iii34. 10.1093/NDT/GFY174 30281132 PMC6168816

[B35] Dai X. Wiernek S. Evans J. P. Runge M. S. (2016). Genetics of coronary artery disease and myocardial infarction. World J. Cardiol. 8, 1–23. 10.4330/WJC.V8.I1.1 26839654 PMC4728103

[B36] Davies G. Armstrong N. Bis J. C. Bressler J. Chouraki V. Giddaluru S. (2015). Genetic contributions to variation in general cognitive function: a meta-analysis of genome-wide association studies in the CHARGE consortium (N = 53949). Mol. Psychiatry 20, 183–192. 10.1038/mp.2014.188 25644384 PMC4356746

[B37] Davyson E. Shen X. Gadd D. A. Bernabeu E. Hillary R. F. McCartney D. L. (2023). Metabolomic investigation of major depressive disorder identifies a potentially causal association with polyunsaturated fatty acids. Biol. Psychiatry 94, 630–639. 10.1016/J.BIOPSYCH.2023.01.027 36764567 PMC10804990

[B38] Dayem Ullah A. Z. Lemoine N. R. Chelala C. (2012). SNPnexus: a web server for functional annotation of novel and publicly known genetic variants (2012 update). Nucleic Acids Res. 40, W65–W70. 10.1093/nar/gks364 22544707 PMC3394262

[B39] Dayem Ullah A. Z. Lemoine N. R. Chelala C. (2013). A practical guide for the functional annotation of genetic variations using SNPnexus. Brief. Bioinform 14, 437–447. 10.1093/bib/bbt004 23395730

[B40] Dayem Ullah A. Z. Oscanoa J. Wang J. Nagano A. Lemoine N. R. Chelala C. (2018). SNPnexus: assessing the functional relevance of genetic variation to facilitate the promise of precision medicine. Nucleic Acids Res. 46, W109-W113–W113. 10.1093/nar/gky399 29757393 PMC6030955

[B41] Deelen J. Beekman M. Uh H. Helmer Q. Kuningas M. Christiansen L. (2011). Genome‐wide association study identifies a single major locus contributing to survival into old age; the APOE locus revisited. Aging Cell 10, 686–698. 10.1111/j.1474-9726.2011.00705.x 21418511 PMC3193372

[B42] Deelen J. Beekman M. Uh H.-W. Broer L. Ayers K. L. Tan Q. (2014). Genome-wide association meta-analysis of human longevity identifies a novel locus conferring survival beyond 90 years of age. Hum. Mol. Genet. 23, 4420–4432. 10.1093/hmg/ddu139 24688116 PMC4103672

[B43] Deloukas P. Kanoni S. Willenborg C. Farrall M. Assimes T. L. Thompson J. R. (2013). Large-scale association analysis identifies new risk loci for coronary artery disease. Nat. Genet. 45, 25–33. 10.1038/ng.2480 23202125 PMC3679547

[B44] Denny J. C. Bastarache L. Ritchie M. D. Carroll R. J. Zink R. Mosley J. D. (2013). Systematic comparison of phenome-wide association study of electronic medical record data and genome-wide association study data. Nat. Biotechnol. 31, 1102–1110. 10.1038/nbt.2749 24270849 PMC3969265

[B45] Dolezal P. Likic V. Tachezy J. Lithgow T. (2006). Evolution of the molecular machines for protein import into mitochondria. Science 313, 314–318. 10.1126/science.1127895 16857931

[B46] Drobni Z. D. Kolossvary M. Karady J. Jermendy A. L. Tarnoki A. D. Tarnoki D. L. (2022). Heritability of coronary artery disease: insights from a classical twin study. Circ. Cardiovasc Imaging 15, e013348. 10.1161/CIRCIMAGING.121.013348 35290075 PMC8925867

[B47] Echouffo-Tcheugui J. B. Jain M. Cheng S. (2020). Breaking through the surface: more to learn about lipids and cardiovascular disease. J. Clin. Investigation 130, 1084–1086. 10.1172/JCI134696 PMC7269560 31985490

[B48] Einarson T. R. Acs A. Ludwig C. Panton U. H. (2018). Prevalence of cardiovascular disease in type 2 diabetes: a systematic literature review of scientific evidence from across the world in 2007–2017. Cardiovasc. Diabetol. 17 (1 17), 83–19. 10.1186/S12933-018-0728-6 29884191 PMC5994068

[B49] Ellis J. Lange E. M. Li J. Dupuis J. Baumert J. Walston J. D. (2014). Large multiethnic Candidate Gene Study for C-reactive protein levels: identification of a novel association at CD36 in African Americans. Hum. Genet. 133, 985–995. 10.1007/s00439-014-1439-z 24643644 PMC4104766

[B50] Elsaid A. Samir eid O. Said S. B. Zahran R. F. (2021). Association of NOS3 (rs 2070744) and SOD2Val16Ala (rs4880) gene polymorphisms with increased risk of ESRD among Egyptian patients. J. Genet. Eng. and Biotechnol. 19, 158. 10.1186/S43141-021-00260-W 34661767 PMC8523625

[B51] Estopinal C. B. Chocron I. M. Parks M. B. Wade E. A. Roberson R. M. Burgess L. G. (2014). Mitochondrial haplogroups are associated with severity of diabetic retinopathy. Invest Ophthalmol. Vis. Sci. 55, 5589–5595. 10.1167/iovs.14-15149 25118268 PMC4160073

[B52] Fan S. Wang Q. Wang A. Y. Zhang P. Zhong X. Chen S. (2021). The association between variants in PLA2R and HLA-DQA1 and renal outcomes in patients with primary membranous nephropathy in Western China. BMC Med. Genomics 14, 123. 10.1186/s12920-021-00969-0 33964912 PMC8105990

[B53] Feitosa M. F. Kraja A. T. Chasman D. I. Sung Y. J. Winkler T. W. Ntalla I. (2018). Novel genetic associations for blood pressure identified via gene-alcohol interaction in up to 570K individuals across multiple ancestries. PLoS One 13, e0198166. 10.1371/JOURNAL.PONE.0198166 29912962 PMC6005576

[B54] Feng Q. Wei W.-Q. Levinson R. T. Mosley J. D. Stein C. M. (2017). Replication and fine-mapping of genetic predictors of lipid traits in African-Americans. J. Hum. Genet. 62, 895–901. 10.1038/jhg.2017.55 28539666 PMC5612856

[B55] Ference B. A. Ginsberg H. N. Graham I. Ray K. K. Packard C. J. Bruckert E. (2017). Low-density lipoproteins cause atherosclerotic cardiovascular disease. 1. Evidence from genetic, epidemiologic, and clinical studies. A consensus statement from the European Atherosclerosis Society Consensus Panel. Eur. Heart J. 38, 2459–2472. 10.1093/eurheartj/ehx144 28444290 PMC5837225

[B56] Foscolou A. Georgousopoulou E. Magriplis E. Naumovski N. Rallidis L. Matalas A. L. (2018). The mediating role of Mediterranean diet on the association between Lp(a) levels and cardiovascular disease risk: a 10-year follow-up of the ATTICA study. Clin. Biochem. 60, 33–37. 10.1016/J.CLINBIOCHEM.2018.07.011 30055165

[B57] Fox C. S. Matsushita K. Woodward M. Bilo H. J. G. Chalmers J. Lambers Heerspink H. J. (2012). Associations of kidney disease measures with mortality and end-stage renal disease in individuals with and without diabetes: a meta-analysis. Lancet 380, 1662–1673. 10.1016/S0140-6736(12)61350-6 23013602 PMC3771350

[B58] Fuku N. Park K. S. Yamada Y. Nishigaki Y. Cho Y. M. Matsuo H. (2007). Mitochondrial haplogroup N9a confers resistance against type 2 diabetes in Asians. Am. J. Hum. Genet. 80, 407–415. 10.1086/512202 17273962 PMC1821119

[B59] Giri A. Hellwege J. N. Keaton J. M. Park J. Qiu C. Warren H. R. (2019). Trans-ethnic association study of blood pressure determinants in over 750,000 individuals. Nat. Genet. 51, 51–62. 10.1038/S41588-018-0303-9 30578418 PMC6365102

[B60] Goff D. C. Lloyd-Jones D. M. Bennett G. Coady S. D’Agostino R. B. Gibbons R. (2014). 2013 ACC/AHA guideline on the assessment of cardiovascular risk: a report of the American college of cardiology/American heart association task force on practice guidelines. Circulation 129, S49–S73. 10.1161/01.cir.0000437741.48606.98 24222018

[B61] Gray M. W. Gray M. W. Burger G. Lang B. F. (2008). Mitochondrial evolution. Science 1476, 1476–1481. 10.1126/science.283.5407.1476 10066161

[B62] Gründemann D. Schömig E. (2000). Gene structures of the human non-neuronal monoamine transporters EMT and OCT2. Hum. Genet. 106, 627–635. 10.1007/S004390000309 10942111

[B63] Gui W. Qiu C. Shao Q. Li J. (2021). Associations of vascular risk factors, APOE and TOMM40 polymorphisms with cognitive function in dementia-free Chinese older adults: a community-based study. Front. Psychiatry 12, 617773. 10.3389/fpsyt.2021.617773 33790814 PMC8005534

[B64] Gunawan A. Fajar J. K. Tamara F. Mahendra A. I. Ilmawan M. Purnamasari Y. (2020). Nitride oxide synthase 3 and klotho gene polymorphisms in the pathogenesis of chronic kidney disease and age-related cognitive impairment: a systematic review and meta-analysis. F1000Res 9, 252. 10.12688/F1000RESEARCH.22989.2 34035901 PMC8112466

[B65] Guo H. Guo L. Yuan Y. Liang X. Y. Bi R. (2022). Co-Occurrence of m.15992A>G and m.15077G>A is associated with a high penetrance of maternally inherited hypertension in a Chinese pedigree. Am. J. Hypertens. 35, 96–102. 10.1093/AJH/HPAB123 34346491

[B66] Guo Y. Lanktree M. B. Taylor K. C. Hakonarson H. Lange L. A. Keating B. J. (2013). Gene-centric meta-analyses of 108 912 individuals confirm known body mass index loci and reveal three novel signals. Hum. Mol. Genet. 22, 184–201. 10.1093/hmg/dds396 23001569 PMC3522401

[B67] Hartiala J. A. Han Y. Jia Q. Hilser J. R. Huang P. Gukasyan J. (2021). Genome-wide analysis identifies novel susceptibility loci for myocardial infarction. Eur. Heart J. 42, 919–933. 10.1093/EURHEARTJ/EHAA1040 33532862 PMC7936531

[B68] Heidari M. M. Mirfakhradini F. S. Tayefi F. Ghorbani S. Khatami M. Hadadzadeh M. (2020). Novel point mutations in mitochondrial MT-CO2 gene may Be risk factors for coronary artery disease. Appl. Biochem. Biotechnol. 191, 1326–1339. 10.1007/S12010-020-03275-0 32096057

[B69] Hoffmann T. J. Ehret G. B. Nandakumar P. Ranatunga D. Schaefer C. Kwok P. Y. (2017). Genome-wide association analyses using electronic health records identify new loci influencing blood pressure variation. Nat. Genet. 49, 54–64. 10.1038/ng.3715 27841878 PMC5370207

[B70] Humphries A. D. Streimann I. C. Stojanovski D. Johnston A. J. Yano M. Hoogenraad N. J. (2005). Dissection of the mitochondrial import and assembly pathway for human Tom40. J. Biol. Chem. 280, 11535–11543. 10.1074/jbc.M413816200 15644312

[B71] Inouye M. Abraham G. Nelson C. P. Wood A. M. Sweeting M. J. Dudbridge F. (2018a). Genomic risk prediction of coronary artery disease in 480,000 adults: implications for primary prevention. J. Am. Coll. Cardiol. 72, 1883–1893. 10.1016/J.JACC.2018.07.079 30309464 PMC6176870

[B72] Inouye M. Abraham G. Nelson C. P. Wood A. M. Sweeting M. J. Dudbridge F. (2018b). Genomic risk prediction of coronary artery disease in nearly 500,000 adults: implications for early screening and primary prevention. bioRxiv, 250712. 10.1101/250712 PMC6176870 30309464

[B73] Jankowski J. Floege J. Fliser D. Böhm M. Marx N. (2021). Cardiovascular disease in chronic kidney disease: pathophysiological insights and therapeutic options. Circulation 143, 1157–1172. 10.1161/CIRCULATIONAHA.120.050686 33720773 PMC7969169

[B74] Kaplan J. (2020). fastDummies: fast creation of dummy (binary) columns and rows from categorical variables. Available at: https://cran.r-project.org/package=fastDummies.

[B75] Khera A. V. Kathiresan S. (2017). Genetics of coronary artery disease: discovery, biology and clinical translation. Nat. Rev. Genet. 18, 331–344. 10.1038/nrg.2016.160 28286336 PMC5935119

[B76] Kichaev G. Bhatia G. Loh P. R. Gazal S. Burch K. Freund M. K. (2019). Leveraging polygenic functional enrichment to improve GWAS power. Am. J. Hum. Genet. 104, 65–75. 10.1016/J.AJHG.2018.11.008 30595370 PMC6323418

[B77] Kim D. S. Park S. (2023). Interactions between polygenetic variants and lifestyle factors in hypothyroidism: a hospital-based cohort study. Nutrients 15, 3850. 10.3390/nu15173850 37686882 PMC10490100

[B78] Kishore P. Kim S. H. Crandall J. P. (2012). Glycemic control and cardiovascular disease: what’s a doctor to do? Curr. Diab Rep. 12, 255–264. 10.1007/S11892-012-0268-5 22467273 PMC3390161

[B79] Kofler B. Mueller E. E. Eder W. Stanger O. Maier R. Weger M. (2009). Mitochondrial DNA haplogroup T is associated with coronary artery disease and diabetic retinopathy: a case control study. BMC Med. Genet. 10, 35. 10.1186/1471-2350-10-35 19383124 PMC2676278

[B80] Koskeridis F. Evangelou E. Said S. Boyle J. J. Elliott P. Dehghan A. (2022). Pleiotropic genetic architecture and novel loci for C-reactive protein levels. Nat. Commun. 13, 6939. 10.1038/S41467-022-34688-6 36376304 PMC9663411

[B81] Köttgen A. Pattaro C. Böger C. A. Fuchsberger C. Olden M. Glazer N. L. (2010). New loci associated with kidney function and chronic kidney disease. Nat. Genet. 42, 376–384. 10.1038/ng.568 20383146 PMC2997674

[B82] Lempiäinen H. Brænne I. Michoel T. Tragante V. Vilne B. Webb T. R. (2018). Network analysis of coronary artery disease risk genes elucidates disease mechanisms and druggable targets. Sci. Rep. 8, 3434. 10.1038/S41598-018-20721-6 29467471 PMC5821758

[B83] Li Q. Liu F. Zheng T. S. Tang J. L. Lu H. J. Jia W. P. (2010). SLC22A2 gene 808 G/T variant is related to plasma lactate concentration in Chinese type 2 diabetics treated with metformin. Acta Pharmacol. Sin. 31, 184–190. 10.1038/APS.2009.189 20139901 PMC4002837

[B84] Liao W.-L. Huang Y.-N. Chang Y.-W. Liu T.-Y. Lu H.-F. Tiao Z.-Y. (2023). Combining polygenic risk scores and human leukocyte antigen variants for personalized risk assessment of type 1 diabetes in the Taiwanese population. Diabetes Obes. Metab. 25, 2928–2936. 10.1111/dom.15187 37455666

[B85] Liao W. L. Liu T. Y. Cheng C. F. Chou Y. P. Wang T. Y. Chang Y. W. (2022). Analysis of HLA variants and Graves’ disease and its comorbidities using a high resolution imputation system to examine electronic medical health records. Front. Endocrinol. (Lausanne) 13, 842673. 10.3389/FENDO.2022.842673 35321340 PMC8936090

[B192] Limas C. J. Limas C. Goldenberg I. F. Blair R. (1995). Possible involvement of the HLA-DQB1 gene in susceptibility and resistance to human dilated cardiomyopathy. Am. Heart. J. 129, 1141–1144. 10.1016/0002-8703(95)90395-X 7754945

[B86] Lin K.-L. Chen S.-D. Lin K.-J. Liou C.-W. Chuang Y.-C. Wang P.-W. (2021). Quality matters? The involvement of mitochondrial quality control in cardiovascular disease. Front. Cell Dev. Biol. 9, 636295. 10.3389/fcell.2021.636295 33829016 PMC8019794

[B193] Littlejohns T. J. Sudlow C. Allen N. E. Collins R. (2019). UK Biobank: opportunities for cardiovascular research. Eur. Heart J. 40, 1158–1166. 10.1093/EURHEARTJ/EHX254 28531320 PMC6451771

[B87] Liu C. Kraja A. T. Smith J. A. Brody J. A. Franceschini N. Bis J. C. (2016). Meta-analysis identifies common and rare variants influencing blood pressure and overlapping with metabolic trait loci. Nat. Genet. 48, 1162–1170. 10.1038/NG.3660 27618448 PMC5320952

[B88] Liu H. Liu X. Zhou J. Li T. (2022). Mitochondrial DNA is a vital driving force in ischemia-reperfusion injury in cardiovascular diseases. Oxid. Med. Cell Longev. 2022, 6235747. 10.1155/2022/6235747 35620580 PMC9129988

[B89] Liu M. Li X.-C. Lu L. Cao Y. Sun R.-R. Chen S. (2014). Cardiovascular disease and its relationship with chronic kidney disease. Eur. Rev. Med. Pharmacol. Sci. 18, 2918–2926. 25339487

[B194] Liu W. Li W. M. Sun N. L. (2005). HLA-DQA1, -DQB1 polymorphism and genetic susceptibility to idiopathic dilated cardiomyopathy in Hans of northern China. Ann. Hum. Genet. 69, 382–388. 10.1111/J.1529-8817.2005.00166.X 15996167

[B90] Liu X. Sun X. Zhang Y. Jiang W. Lai M. Wiggins K. L. (2023). Association between whole blood–derived mitochondrial DNA copy number, low‐density lipoprotein cholesterol, and cardiovascular disease risk. J. Am. Heart Assoc. 12, e029090. 10.1161/JAHA.122.029090 37804200 PMC10757530

[B91] Liu Y. Ma H. Zhu Q. Zhang B. Yan H. Li H. (2019). A genome-wide association study on lipoprotein (a) levels and coronary artery disease severity in a Chinese population. J. Lipid Res. 60, 1440–1448. 10.1194/JLR.P091009 31186284 PMC6672037

[B92] Lodish H. Berk A. Zipursky S. Lawrence Matsudaira P. Baltimore D. Darnell J. (2012). Electron transport and oxidative phosphorylation. Mol. Cell Biol. 474. 10.1016/S1470-8175(01)00023-6

[B93] Lopez E. O. Ballard B. D. Jan A. (2023). Cardiovascular disease. Nursing. *(Brux)* .

[B94] Lotta L. A. Pietzner M. Stewart I. D. Wittemans L. B. L. Li C. Bonelli R. (2021). A cross-platform approach identifies genetic regulators of human metabolism and health. Nat. Genet. 53, 54–64. 10.1038/S41588-020-00751-5 33414548 PMC7612925

[B95] Ma Z.-J. Sun P. Guo G. Zhang R. Chen L.-M. (2013). Association of the HLA-DQA1 and HLA-DQB1 alleles in type 2 diabetes mellitus and diabetic nephropathy in the han ethnicity of China. J. Diabetes Res. 2013, 452537. 10.1155/2013/452537 23671871 PMC3647553

[B96] Mack S. Coassin S. Rueedi R. Yousri N. A. Seppälä I. Gieger C. (2017). A genome-wide association meta-analysis on lipoprotein (a) concentrations adjusted for apolipoprotein (a) isoforms. J. Lipid Res. 58, 1834–1844. 10.1194/jlr.M076232 28512139 PMC5580897

[B97] Mahmoodi B. K. Matsushita K. Woodward M. Blankestijn P. J. Cirillo M. Ohkubo T. (2012). Associations of kidney disease measures with mortality and end-stage renal disease in individuals with and without hypertension: a meta-analysis. Lancet 380, 1649–1661. 10.1016/S0140-6736(12)61272-0 23013600 PMC3993095

[B98] Marino B. Digilio M. C. (2000). Congenital heart disease and genetic syndromes: specific correlation between cardiac phenotype and genotype. Cardiovasc Pathol. 9, 303–315. 10.1016/s1054-8807(00)00050-8 11146300

[B99] Medina A. M. Zubero E. E. Jiménez M. A. A. Barragan S. A. A. García C. A. L. Ramos J. J. G. (2018). NOS3 polymorphisms and chronic kidney disease. J. Bras. Nefrol. 40, 273–277. 10.1590/2175-8239-JBN-3824 29927456 PMC6533959

[B100] Meiklejohn C. D. Holmbeck M. A. Siddiq M. A. Abt D. N. Rand D. M. Montooth K. L. (2013). An incompatibility between a mitochondrial tRNA and its nuclear-encoded tRNA synthetase compromises development and fitness in Drosophila. PLoS Genet. 9, e1003238. 10.1371/journal.pgen.1003238 23382693 PMC3561102

[B101] Miao L. Yin R.-X. Pan S.-L. Yang S. Yang D.-Z. Lin W.-X. (2018). BCL3-PVRL2-TOMM40 SNPs, gene-gene and gene-environment interactions on dyslipidemia. Sci. Rep. 8, 6189. 10.1038/s41598-018-24432-w 29670124 PMC5906470

[B102] Middelberg R. P. Ferreira M. A. Henders A. K. Heath A. C. Madden P. A. Montgomery G. W. (2011). Genetic variants in LPL, OASL and TOMM40/APOE-C1-C2-C4 genes are associated with multiple cardiovascular-related traits. BMC Med. Genet. 12, 123. 10.1186/1471-2350-12-123 21943158 PMC3189113

[B103] Muntean I. Togănel R. Benedek T. (2017). Genetics of congenital heart disease: past and present. Biochem. Genet. 55, 105–123. 10.1007/s10528-016-9780-7 27807680

[B104] National Institute for Health and Care Excellence [NICE] (2017). Hypertension in adults: diagnosis and management. Evidence review for targets NICE guideline NG136. 31577399

[B105] National Institute for Health and Care Excellenve (NICE) (2019). Hypertension in adults: diagnosis and management. 31577399

[B106] Nazarian A. Yashin A. I. Kulminski A. M. (2019). Genome-wide analysis of genetic predisposition to Alzheimer’s disease and related sex disparities. Alzheimers Res. Ther. 11, 5. 10.1186/s13195-018-0458-8 30636644 PMC6330399

[B107] Nikpay M. Goel A. Won H. H. Hall L. M. Willenborg C. Kanoni S. (2015). A comprehensive 1,000 Genomes-based genome-wide association meta-analysis of coronary artery disease. Nat. Genet. 47, 1121–1130. 10.1038/NG.3396 26343387 PMC4589895

[B108] Nishigaki Y. Yamada Y. Fuku N. Matsuo H. Segawa T. Watanabe S. (2007a). Mitochondrial haplogroup A is a genetic risk factor for atherothrombotic cerebral infarction in Japanese females. Mitochondrion 7, 72–79. 10.1016/j.mito.2006.11.002 17257906

[B109] Nishigaki Y. Yamada Y. Fuku N. Matsuo H. Segawa T. Watanabe S. (2007b). Mitochondrial haplogroup N9b is protective against myocardial infarction in Japanese males. Hum. Genet. 120, 827–836. 10.1007/s00439-006-0269-z 17033820

[B110] Onengut-Gumuscu S. Chen W.-M. Robertson C. C. Bonnie J. K. Farber E. Zhu Z. (2019). Type 1 diabetes risk in african-ancestry participants and utility of an ancestry-specific genetic risk score. Diabetes Care 42, 406–415. 10.2337/dc18-1727 30659077 PMC6385701

[B111] Oscanoa J. Sivapalan L. Gadaleta E. Dayem Ullah A. Z. Lemoine N. R. Chelala C. (2020). SNPnexus: a web server for functional annotation of human genome sequence variation (2020 update). Nucleic Acids Res. 48, W185-W192–W192. 10.1093/NAR/GKAA420 32496546 PMC7319579

[B112] Ouyang J. Wang H. Huang J. (2023). The role of lactate in cardiovascular diseases. Cell Commun. Signal. 21 (1), 317–414. 10.1186/S12964-023-01350-7 37924124 PMC10623854

[B113] Padhi U. N. Mulkalwar M. Saikrishna L. Verma H. K. Bhaskar L. (2022). NOS3 gene intron 4 a/b polymorphism is associated with ESRD in autosomal dominant polycystic kidney disease patients. J. Bras. Nefrol. 44, 224–231. 10.1590/2175-8239-JBN-2021-0089 35138322 PMC9269174

[B114] Pagliarini D. J. Calvo S. E. Chang B. Sheth S. A. Vafai S. B. Ong S.-E. (2008). A mitochondrial protein compendium elucidates complex I disease biology. Cell 134, 112–123. 10.1016/j.cell.2008.06.016 18614015 PMC2778844

[B115] Palacín M. Alvarez V. Martín M. Díaz M. Corao A. I. Alonso B. (2011). Mitochondrial DNA and TFAM gene variation in early-onset myocardial infarction: evidence for an association to haplogroup H. Mitochondrion 11, 176–181. 10.1016/j.mito.2010.09.004 20863902

[B116] Palmer N. D. Kahali B. Kuppa A. Chen Y. Du X. Feitosa M. F. (2021). Allele-specific variation at APOE increases nonalcoholic fatty liver disease and obesity but decreases risk of Alzheimer’s disease and myocardial infarction. Hum. Mol. Genet. 30, 1443–1456. 10.1093/hmg/ddab096 33856023 PMC8283205

[B117] Pattaro C. Teumer A. Gorski M. Chu A. Y. Li M. Mijatovic V. (2016). Genetic associations at 53 loci highlight cell types and biological pathways relevant for kidney function. Nat. Commun. 7, 10023. 10.1038/ncomms10023 26831199 PMC4735748

[B118] Piotrowska-Nowak A. Elson J. L. Sobczyk-Kopciol A. Piwonska A. Puch-Walczak A. Drygas W. (2018). New mtDNA association model, MutPred variant load, suggests individuals with multiple mildly deleterious mtDNA variants are more likely to suffer from atherosclerosis. Front. Genet. 9, 702. 10.3389/fgene.2018.00702 30671084 PMC6332467

[B119] Plotnikov D. Huang Y. Khawaja A. P. Foster P. J. Zhu Z. Guggenheim J. A. (2022). High blood pressure and intraocular pressure: a mendelian randomization study. Invest Ophthalmol. Vis. Sci. 63, 29. 10.1167/IOVS.63.6.29 PMC9251815 35762941

[B120] Poznyak A. V. Ivanova E. A. Sobenin I. A. Yet S. F. Orekhov A. N. (2020). The role of mitochondria in cardiovascular diseases. Biol. (Basel) 9 (6), 137. 10.3390/BIOLOGY9060137 PMC7344641 32630516

[B121] Qian J. Chen Y. Yang X. Wang Q. Zhao J. Deng X. (2023). Association study identified HLA-DQA1 as a novel genetic risk of systemic lupus erythematosus-associated pulmonary arterial hypertension. Arthritis Rheumatol. 75, 2207–2215. 10.1002/art.42641 37382296

[B122] Radovica I. Fridmanis D. Silamikelis I. Nikitina-Zake L. Klovins J. (2014). Association between CETP, MLXIPL, and TOMM40 polymorphisms and serum lipid levels in a Latvian population. Meta Gene 2, 565–578. 10.1016/j.mgene.2014.07.006 25606439 PMC4287865

[B123] Rai H. Fitzgerald S. Coughlan J. J. Spence M. Colleran R. Joner M. (2023). Glu298Asp variant of the endothelial nitric oxide synthase gene and acute coronary syndrome or premature coronary artery disease: a systematic review and meta-analysis. Nitric Oxide 138–139, 85–95. 10.1016/J.NIOX.2023.07.001 37451608

[B124] Ray D. Chatterjee N. (2020). A powerful method for pleiotropic analysis under composite null hypothesis identifies novel shared loci between Type 2 Diabetes and Prostate Cancer. PLoS Genet. 16, e1009218. 10.1371/JOURNAL.PGEN.1009218 33290408 PMC7748289

[B125] R Core Team (2024). R: A Language and Environment for Statistical Computing. Vienna: R Foundation for Statistical Computing. Available at: https://www.r-project.org/.

[B195] Razieh C. Zaccardi F. Miksza J. Davies M. J. Hansell A. L. Khunti K. (2022). Differences in the risk of cardiovascular disease across ethnic groups: UK Biobank observational study. Nutr. Metab. Cardiovasc. Dis. 32, 2594–2602. 10.1016/J.NUMECD.2022.08.002 36064688

[B126] Richardson T. G. Leyden G. M. Wang Q. Bell J. A. Elsworth B. Smith G. D. (2022). Characterising metabolomic signatures of lipid-modifying therapies through drug target mendelian randomisation. PLoS Biol. 20, e3001547. 10.1371/JOURNAL.PBIO.3001547 35213538 PMC8906647

[B127] Rosa A. Fonseca B. V. Krug T. Manso H. Gouveia L. Albergaria I. (2008). Mitochondrial haplogroup H1 is protective for ischemic stroke in Portuguese patients. BMC Med. Genet. 9, 57. 10.1186/1471-2350-9-57 18593462 PMC2492853

[B128] Roumeliotis A. Roumeliotis S. Tsetsos F. Georgitsi M. Georgianos P. I. Stamou A. (2021). Oxidative stress genes in diabetes mellitus type 2: association with diabetic kidney disease. Oxid. Med. Cell Longev. 2021, 2531062. 10.1155/2021/2531062 34545296 PMC8448992

[B129] Safdar M. Ullah M. Wahab A. Hamayun S. Ur Rehman M. Khan M. A. (2024). Genomic insights into heart health: exploring the genetic basis of cardiovascular disease. Curr. Probl. Cardiol. 49, 102182. 10.1016/j.cpcardiol.2023.102182 37913933

[B130] Sakaue S. Kanai M. Tanigawa Y. Karjalainen J. Kurki M. Koshiba S. (2021). A cross-population atlas of genetic associations for 220 human phenotypes. Nat. Genet. 53, 1415–1424. 10.1038/S41588-021-00931-X 34594039 PMC12208603

[B131] Salakhov R. R. Goncharova I. A. Makeeva O. A. Golubenko M. V. Kulish E. V. Kashtalap V. V. (2014). TOMM40 gene polymorphisms association with lipid profile. Russ. J. Genet. 50, 198–204. 10.1134/S1022795413120090 25711031

[B132] Sandhu M. S. Waterworth D. M. Debenham S. L. Wheeler E. Papadakis K. Zhao J. H. (2008). LDL-cholesterol concentrations: a genome-wide association study. Lancet 371, 483–491. 10.1016/S0140-6736(08)60208-1 18262040 PMC2292820

[B133] Sawabe M. Tanaka M. Chida K. Arai T. Nishigaki Y. Fuku N. (2011). Mitochondrial haplogroups A and M7a confer a genetic risk for coronary atherosclerosis in the Japanese elderly: an autopsy study of 1,536 patients. J. Atheroscler. Thromb. 18, 166–175. 10.5551/jat.6742 21099167

[B134] Schatz U. Tselmin S. Müller G. Julius U. Hohenstein B. Fischer S. (2017). Most significant reduction of cardiovascular events in patients undergoing lipoproteinapheresis due to raised Lp(a) levels - a multicenter observational study. Atheroscler. Suppl. 30, 246–252. 10.1016/J.ATHEROSCLEROSISSUP.2017.05.047 29096845

[B135] Schoeler T. Speed D. Porcu E. Pirastu N. Pingault J. B. Kutalik Z. (2023). Participation bias in the UK Biobank distorts genetic associations and downstream analyses. Nat. Hum. Behav. 7, 1216–1227. 10.1038/S41562-023-01579-9 37106081 PMC10365993

[B136] Scott R. A. Scott L. J. Mägi R. Marullo L. Gaulton K. J. Kaakinen M. (2017). An expanded genome-wide association study of type 2 diabetes in Europeans. Diabetes 66, 2888–2902. 10.2337/db16-1253 28566273 PMC5652602

[B137] Shadrina A. S. Shashkova T. I. Torgasheva A. A. Sharapov S. Z. Klarić L. Pakhomov E. D. (2020). Prioritization of causal genes for coronary artery disease based on cumulative evidence from experimental and *in silico* studies. Sci. Rep. 10, 10486. 10.1038/S41598-020-67001-W 32591598 PMC7320185

[B138] Shen C. Guo Z. Wu M. Hu X. Yang G. Yu R. (2007). Association study between hypertension and A/G polymorphism at codon 637 of the transporter associated with antigen processing 1 gene. Hypertens. Res. 30, 683–690. 10.1291/hypres.30.683 17917315

[B139] Siasos G. Tsigkou V. Kosmopoulos M. Theodosiadis D. Simantiris S. Tagkou N. M. (2018). Mitochondria and cardiovascular diseases-from pathophysiology to treatment. Ann. Transl. Med. 6, 256. 10.21037/atm.2018.06.21 30069458 PMC6046286

[B140] Silva S. Nitsch D. Fatumo S. (2023). Genome-wide association studies on coronary artery disease: a systematic review and implications for populations of different ancestries. PLoS One 18, e0294341. 10.1371/JOURNAL.PONE.0294341 38019802 PMC10686512

[B141] Singh L. N. Ennis B. Loneragan B. Tsao N. L. Lopez Sanchez M. I. G. Li J. (2021). MitoScape: a big-data, machine-learning platform for obtaining mitochondrial DNA from next-generation sequencing data. PLoS Comput. Biol. 17, e1009594. 10.1371/journal.pcbi.1009594 34762648 PMC8610268

[B142] Sinnott-Armstrong N. Tanigawa Y. Amar D. Mars N. Benner C. Aguirre M. (2021). Genetics of 35 blood and urine biomarkers in the UK Biobank. Nat. Genet. 53, 185–194. 10.1038/s41588-020-00757-z 33462484 PMC7867639

[B143] Skelly R. (2020). Next generation sequencing and genome-wide association studies to identify mitochondrial genomic features associated with diabetic kidney disease.

[B144] Skol A. D. Scott L. J. Abecasis G. R. Boehnke M. (2006). Joint analysis is more efficient than replication-based analysis for two-stage genome-wide association studies. Nat. Genet. 38, 209–213. 10.1038/ng1706 16415888

[B145] Smith A. C. Robinson A. J. (2016). MitoMiner v3.1, an update on the mitochondrial proteomics database. Nucleic Acids Res. 44, D1258–D1261. 10.1093/nar/gkv1001 26432830 PMC4702766

[B146] Sung Y. J. Winkler T. W. de las Fuentes L. Bentley A. R. Brown M. R. Kraja A. T. (2018). A large-scale multi-ancestry genome-wide study accounting for smoking behavior identifies multiple significant loci for blood pressure. Am. J. Hum. Genet. 102, 375–400. 10.1016/J.AJHG.2018.01.015 29455858 PMC5985266

[B147] Svishcheva G. R. Belonogova N. M. Zorkoltseva I. V. Kirichenko A. V. Axenovich T. I. (2019). Gene-based association tests using GWAS summary statistics. Bioinformatics 35, 3701–3708. 10.1093/BIOINFORMATICS/BTZ172 30860568

[B148] Taanman J.-W. (1999). The mitochondrial genome: structure, transcription, translation and replication. Biochimica Biophysica Acta (BBA) - Bioenergetics 1410, 103–123. 10.1016/S0005-2728(98)00161-3 10076021

[B149] Talmud P. J. Drenos F. Shah S. Shah T. Palmen J. Verzilli C. (2009). Gene-centric association signals for lipids and apolipoproteins identified via the HumanCVD BeadChip. Am. J. Hum. Genet. 85, 628–642. 10.1016/j.ajhg.2009.10.014 19913121 PMC2775832

[B150] Taylor S. W. Fahy E. Zhang B. Glenn G. M. Warnock D. E. Wiley S. (2003). Characterization of the human heart mitochondrial proteome. Nat. Biotechnol. 21, 281–286. 10.1038/nbt793 12592411

[B151] Temprano-Sagrera G. Sitlani C. M. Bone W. P. Martin-Bornez M. Voight B. F. Morrison A. C. (2022). Multi-phenotype analyses of hemostatic traits with cardiovascular events reveal novel genetic associations. J. Thromb. Haemost. 20, 1331–1349. 10.1111/JTH.15698 35285134 PMC9314075

[B152] The UniProt Consortium (2017). UniProt: the universal protein knowledgebase. Nucleic Acids Res. 45, D158–D169. 10.1093/nar/gkw1099 27899622 PMC5210571

[B153] Timmis J. N. Ayliffe M. A. Huang C. Y. Martin W. (2004). Endosymbiotic gene transfer: organelle genomes forge eukaryotic chromosomes. Nat. Rev. Genet. 5, 123–135. 10.1038/nrg1271 14735123

[B154] Tipping R. W. Ford C. E. Simpson L. M. Walldius G. Jungner I. Folsom A. R. (2009). Lipoprotein(a) concentration and the risk of coronary heart disease, stroke, and nonvascular mortality. JAMA J. Am. Med. Assoc. 302, 412–423. 10.1001/JAMA.2009.1063 PMC3272390 19622820

[B155] Torres G. G. Dose J. Hasenbein T. P. Nygaard M. Krause-Kyora B. Mengel-From J. (2022). Long-lived individuals show a lower burden of variants predisposing to age-related diseases and a higher polygenic longevity score. Int. J. Mol. Sci. 23, 10949. 10.3390/ijms231810949 36142858 PMC9504529

[B156] Tsai M.-H. Kuo C.-W. Lin T.-K. Ho C.-J. Wang P.-W. Chuang J.-H. (2020). Ischemic stroke risk associated with mitochondrial haplogroup F in the asian population. Cells 9, 1885. 10.3390/cells9081885 32796743 PMC7463505

[B157] Tsao C. W. Aday A. W. Almarzooq Z. I. Anderson C. A. M. Arora P. Avery C. L. (2023). Heart disease and stroke statistics-2023 update: a report from the American heart association. Circulation 147, e93–e621. 10.1161/CIR.0000000000001123 36695182 PMC12135016

[B158] Van Der Harst P. Verweij N. (2018). Identification of 64 novel genetic loci provides an expanded view on the genetic architecture of coronary artery disease. Circ. Res. 122, 433–443. 10.1161/CIRCRESAHA.117.312086 29212778 PMC5805277

[B159] van Oven M. (2015). PhyloTree Build 17: growing the human mitochondrial DNA tree. Forensic Sci. Int. Genet. Suppl. Ser. 5, e392–e394. 10.1016/j.fsigss.2015.09.155

[B160] Vázquez P. Hernández-Sánchez C. Escalona-Garrido C. Pereira L. Contreras C. López M. (2018). Increased FGF21 in brown adipose tissue of tyrosine hydroxylase heterozygous mice: implications for cold adaptation. J. Lipid Res. 59, 2308–2320. 10.1194/jlr.M085209 30352954 PMC6277155

[B161] Venter M. Malan L. van Dyk E. Elson J. L. van der Westhuizen F. H. (2017). Using MutPred derived mtDNA load scores to evaluate mtDNA variation in hypertension and diabetes in a two-population cohort: the SABPA study. J. Genet. Genomics 44, 139–149. 10.1016/j.jgg.2016.12.003 28298255

[B162] Venter M. Van Der Westhuizen F. H. Elson J. L. (2018). The aetiology of cardiovascular disease: a role formitochondrial DNA? Cardiovasc J. Afr. 29, 122–132. 10.5830/CVJA-2017-037 28906532 PMC6009096

[B163] Veronese N. Stubbs B. Koyanagi A. Vaona A. Demurtas J. Schofield P. (2019). Mitochondrial genetic haplogroups and cardiovascular diseases: data from the Osteoarthritis Initiative. PLoS One 14, e0213656. 10.1371/journal.pone.0213656 30921349 PMC6438497

[B164] Visseren F. L. J. Mach F. Smulders Y. M. Carballo D. Koskinas K. C. Bäck M. (2021). 2021 ESC Guidelines on cardiovascular disease prevention in clinical practice. Eur. Heart J. 42, 3227–3337. 10.1093/eurheartj/ehab484 34458905

[B165] Wain H. M. Bruford E. A. Lovering R. C. Lush M. J. Wright M. W. Povey S. (2002). Guidelines for human gene nomenclature. Genomics 79, 464–470. 10.1006/geno.2002.6748 11944974

[B166] Wain L. V. Vaez A. Jansen R. Joehanes R. Van Der Most P. J. Erzurumluoglu A. M. (2017). Novel blood pressure locus and gene discovery using genome-wide association study and expression data sets from blood and the kidney. Hypertension 70, e4–e19. 10.1161/HYPERTENSIONAHA.117.09438 28739976 PMC5783787

[B167] Wang Z. Chen H. Qin M. Liu C. Ma Q. Chen X. (2021). Associations of mitochondrial variants with lipidomic traits in a Chinese cohort with coronary artery disease. Front. Genet. 12, 630359. 10.3389/fgene.2021.630359 33841498 PMC8027325

[B168] Warrens H. Banerjee D. Herzog C. A. (2022). Cardiovascular complications of chronic kidney disease: an introduction. Eur. Cardiol. Rev. 17, e13. 10.15420/ECR.2021.54 PMC9127633 35620357

[B169] Watanabe K. Taskesen E. van Bochoven A. Posthuma D. (2017). Functional mapping and annotation of genetic associations with FUMA. Nat. Commun. 8, 1826. 10.1038/s41467-017-01261-5 29184056 PMC5705698

[B170] Watanabe K. Umićević Mirkov M. de Leeuw C. A. van den Heuvel M. P. Posthuma D. (2019). Genetic mapping of cell type specificity for complex traits. Nat. Commun. 10, 3222. 10.1038/s41467-019-11181-1 31324783 PMC6642112

[B171] Watkins H. Farrall M. (2006). Genetic susceptibility to coronary artery disease: from promise to progress. Nat. Rev. Genet. 7, 163–173. 10.1038/nrg1805 16462853

[B172] Wb K. Dl M. (1979). Diabetes and cardiovascular disease. The Framingham study. JAMA 241, 2035–2038. 10.1001/JAMA.241.19.2035 430798

[B173] Weissensteiner H. Pacher D. Kloss-Brandstätter A. Forer L. Specht G. Bandelt H.-J. (2016). HaploGrep 2: mitochondrial haplogroup classification in the era of high-throughput sequencing. Nucleic Acids Res. 44, W58–W63. 10.1093/nar/gkw233 27084951 PMC4987869

[B174] Whelton P. K. Carey R. M. Aronow W. S. Casey D. E. Collins K. J. Dennison Himmelfarb C. (2018). 2017 ACC/AHA/AAPA/ABC/ACPM/AGS/APhA/ASH/ASPC/NMA/PCNA guideline for the prevention, detection, evaluation, and management of high blood pressure in adults: a report of the American college of cardiology/American heart association task force on clinical practice guidelines. J. Am. Coll. Cardiol. 71, e127–e248. 10.1016/j.jacc.2017.11.006 29146535

[B175] WHO (2023). The top 10 causes of death. Available at: https://www.who.int/news-room/fact-sheets/detail/the-top-10-causes-of-death (Accessed December 28, 2023).

[B176] Wojcik G. L. Graff M. Nishimura K. K. Tao R. Haessler J. Gignoux C. R. (2019). Genetic analyses of diverse populations improves discovery for complex traits. Nature 570, 514–518. 10.1038/S41586-019-1310-4 31217584 PMC6785182

[B177] Wuttke M. Wong C. S. Wuhl E. Epting D. Luo L. Hoppmann A. (2016). Genetic loci associated with renal function measures and chronic kidney disease in children: the Pediatric Investigation for Genetic Factors Linked with Renal Progression Consortium. Nephrol. Dial. Transpl. 31, 262–269. 10.1093/ndt/gfv342 PMC4829056 26420894

[B178] Xue A. Wu Y. Zhu Z. Zhang F. Kemper K. E. Zheng Z. (2018). Genome-wide association analyses identify 143 risk variants and putative regulatory mechanisms for type 2 diabetes. Nat. Commun. 9, 2941. 10.1038/S41467-018-04951-W 30054458 PMC6063971

[B179] Yan Q. Nho K. Del-Aguila J. L. Wang X. Risacher S. L. Fan K.-H. (2021). Genome-wide association study of brain amyloid deposition as measured by Pittsburgh Compound-B (PiB)-PET imaging. Mol. Psychiatry 26, 309–321. 10.1038/s41380-018-0246-7 30361487 PMC6219464

[B180] Yang J. Guo Q. Feng X. Liu Y. Zhou Y. (2022). Mitochondrial dysfunction in cardiovascular diseases: potential targets for treatment. Front. Cell Dev. Biol. 10, 841523. 10.3389/fcell.2022.841523 35646910 PMC9140220

[B181] Yang R. Xu H. Pedersen N. L. Li X. Yu J. Bao C. (2021). A healthy lifestyle mitigates the risk of heart disease related to type 2 diabetes: a prospective nested case–control study in a nationwide Swedish twin cohort. Diabetologia 64, 530–539. 10.1007/s00125-020-05324-z 33169206 PMC7864843

[B182] Yeo A. Li L. Warren L. Aponte J. Fraser D. King K. (2017). Pharmacogenetic meta-analysis of baseline risk factors, pharmacodynamic, efficacy and tolerability endpoints from two large global cardiovascular outcomes trials for darapladib. PLoS One 12, e0182115. 10.1371/JOURNAL.PONE.0182115 28753643 PMC5533343

[B183] Yonova-Doing E. Calabrese C. Gomez-Duran A. Schon K. Wei W. Karthikeyan S. (2021). An atlas of mitochondrial DNA genotype–phenotype associations in the UK Biobank. Nat. Genet. 53 (7), 982–993. 10.1038/S41588-021-00868-1 34002094 PMC7611844

[B184] Zerbino D. R. Achuthan P. Akanni W. Amode M. R. Barrell D. Bhai J. (2018). Ensembl 2018. Nucleic Acids Res. 46, D754-D761–D761. 10.1093/nar/gkx1098 29155950 PMC5753206

[B196] Zhang S. Jiang Z. Zhang H. Liu Y. Qi J. Yan Y. (2024). Association of cigarette smoking, smoking cessation with the risk of cardiometabolic multimorbidity in the UK Biobank. BMC Public Health 24, 1910. 10.1186/S12889-024-19457-Y 39014423 PMC11253396

[B185] Zhang Y. Tsai T.-H. Ezrokhi M. Stoelzel C. Cincotta A. H. (2023). Tyrosine hydroxylase knockdown at the hypothalamic supramammillary nucleus area induces obesity and glucose intolerance. Neuroendocrinology 114, 483–510. 10.1159/000535944 38128505 PMC11098027

[B186] Zhao W. Rasheed A. Tikkanen E. Lee J. J. Butterworth A. S. Howson J. M. M. (2017). Identification of new susceptibility loci for type 2 diabetes and shared etiological pathways with coronary heart disease. Nat. Genet. 49, 1450–1457. 10.1038/NG.3943 28869590 PMC5844224

[B187] Zhelankin A. V. Sazonova M. A. Khasanova Z. B. Sinyov V. V. Mitrofanov K. Y. Sobenin I. A. (2015). Analysis of mitochondrial haplogroups in persons with subclinical atherosclerosis based on high-throughput mtDNA sequencing. Patol. Fiziol. Eksp. Ter. 59, 12–16. 26226684

[B188] Zhou W. Nielsen J. B. Fritsche L. G. Dey R. Gabrielsen M. E. Wolford B. N. (2018). Efficiently controlling for case-control imbalance and sample relatedness in large-scale genetic association studies. Nat. Genet. 50 (9 50), 1335–1341. 10.1038/s41588-018-0184-y 30104761 PMC6119127

[B189] Zhu Y. Gu X. Xu C. (2016). A mitochondrial DNA A8701G mutation associated with maternally inherited hypertension and dilated cardiomyopathy in a Chinese pedigree of a consanguineous marriage. Chin. Med. J. Engl. 129, 259–266. 10.4103/0366-6999.174491 26831225 PMC4799567

[B190] Zhu Y. Gu X. Xu C. (2018). Mitochondrial DNA 7908–8816 region mutations in maternally inherited essential hypertensive subjects in China. BMC Med. Genomics 11, 89. 10.1186/S12920-018-0408-0 30326913 PMC6191914

[B191] Zoccali C. Mark P. B. Sarafidis P. Agarwal R. Adamczak M. Bueno de Oliveira R. (2023). Diagnosis of cardiovascular disease in patients with chronic kidney disease. Nat. Rev. Nephrol. 2023 19 (11 19), 733–746. 10.1038/s41581-023-00747-4 37612381

