# Supplementary figures and images for: Mitochondrial related variants associated with cardiovascular traits
Source: Front Physiol. 2024 Aug 27;15:1395371. doi: 10.3389/fphys.2024.1395371 (PMC11385366; doi:10.3389/fphys.2024.1395371)

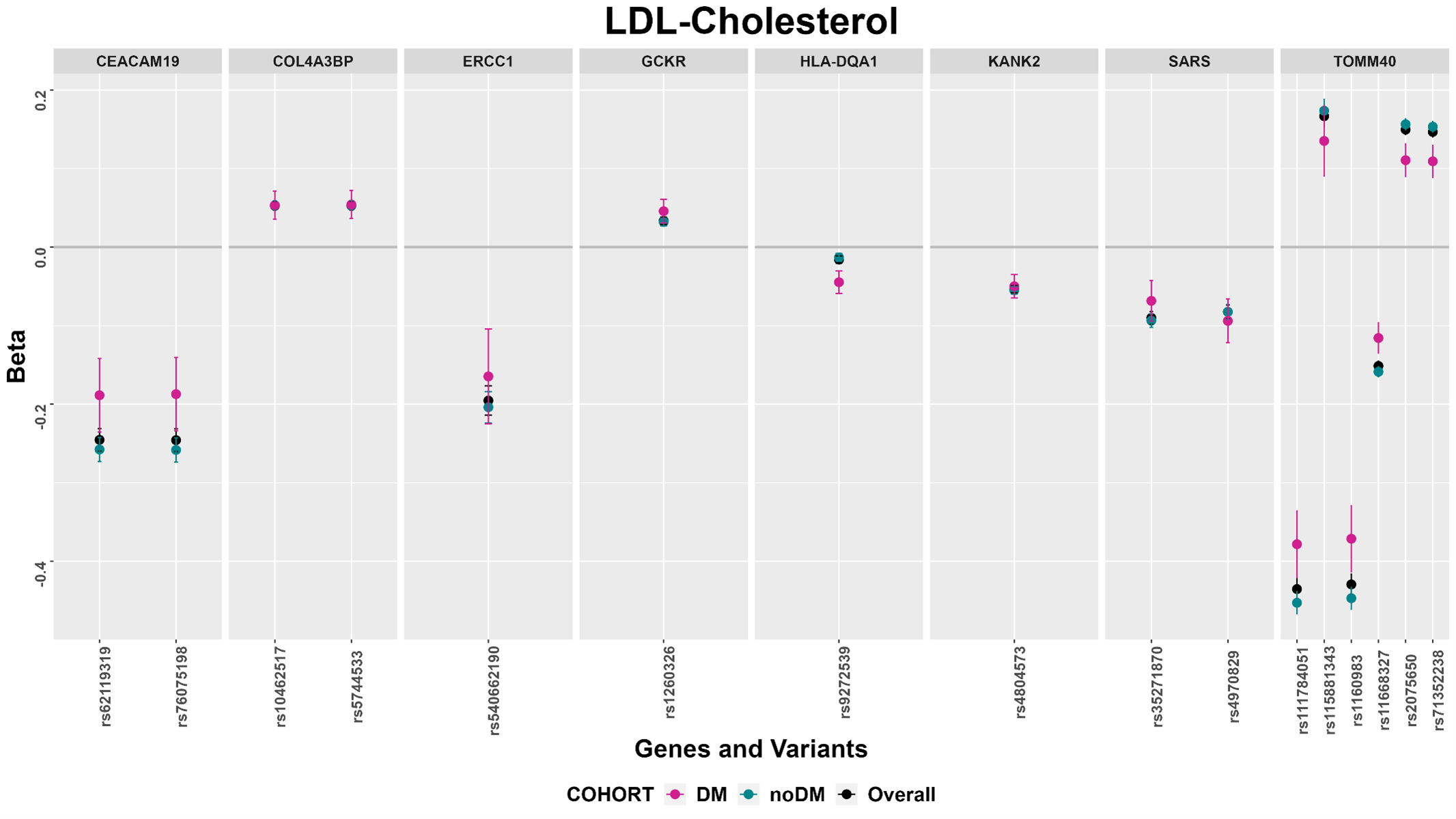

Supplement: Supplementary file 4 [file Image3.TIF]

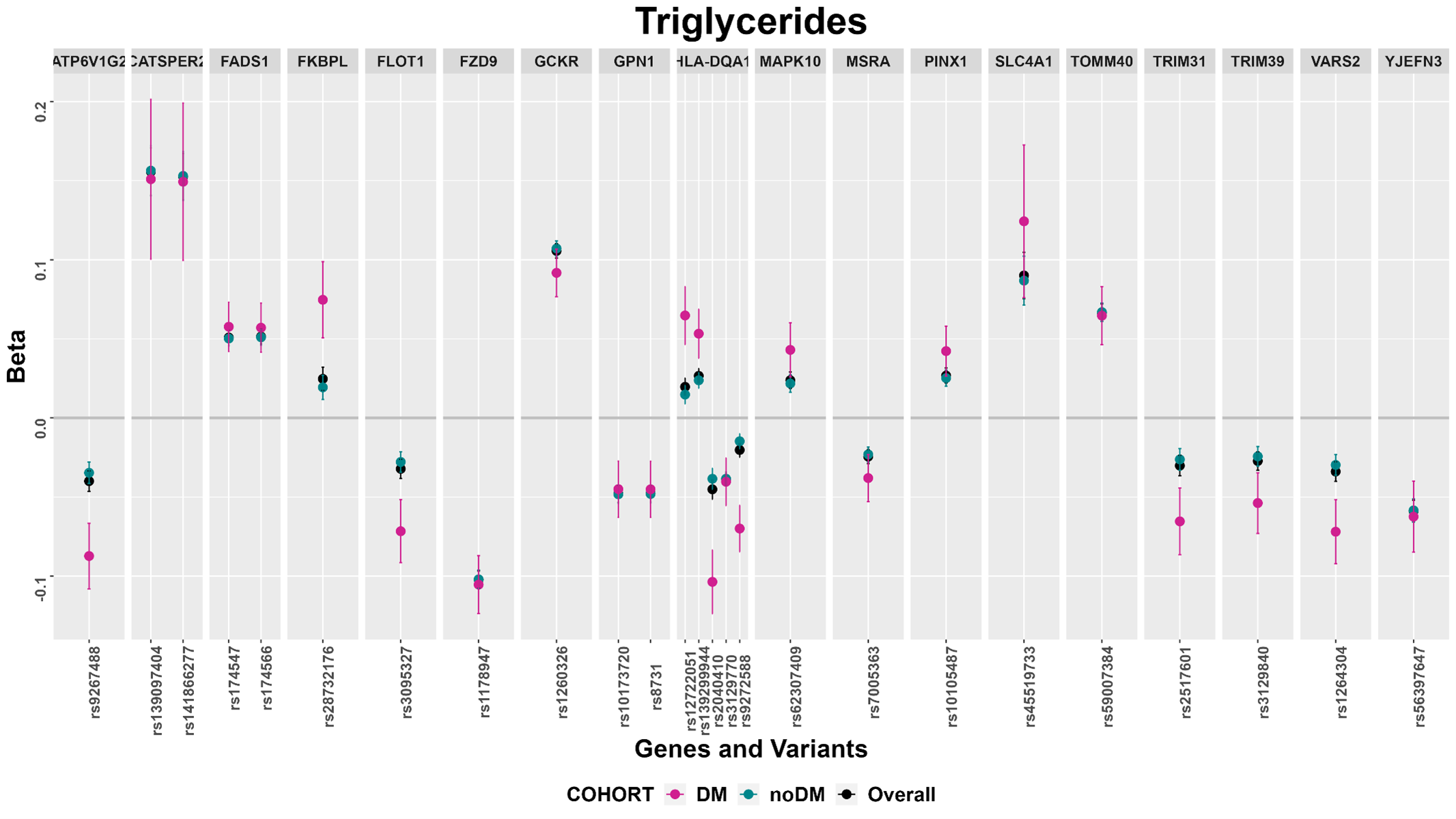

Supplement: Supplementary file 5 [file Image4.TIF]

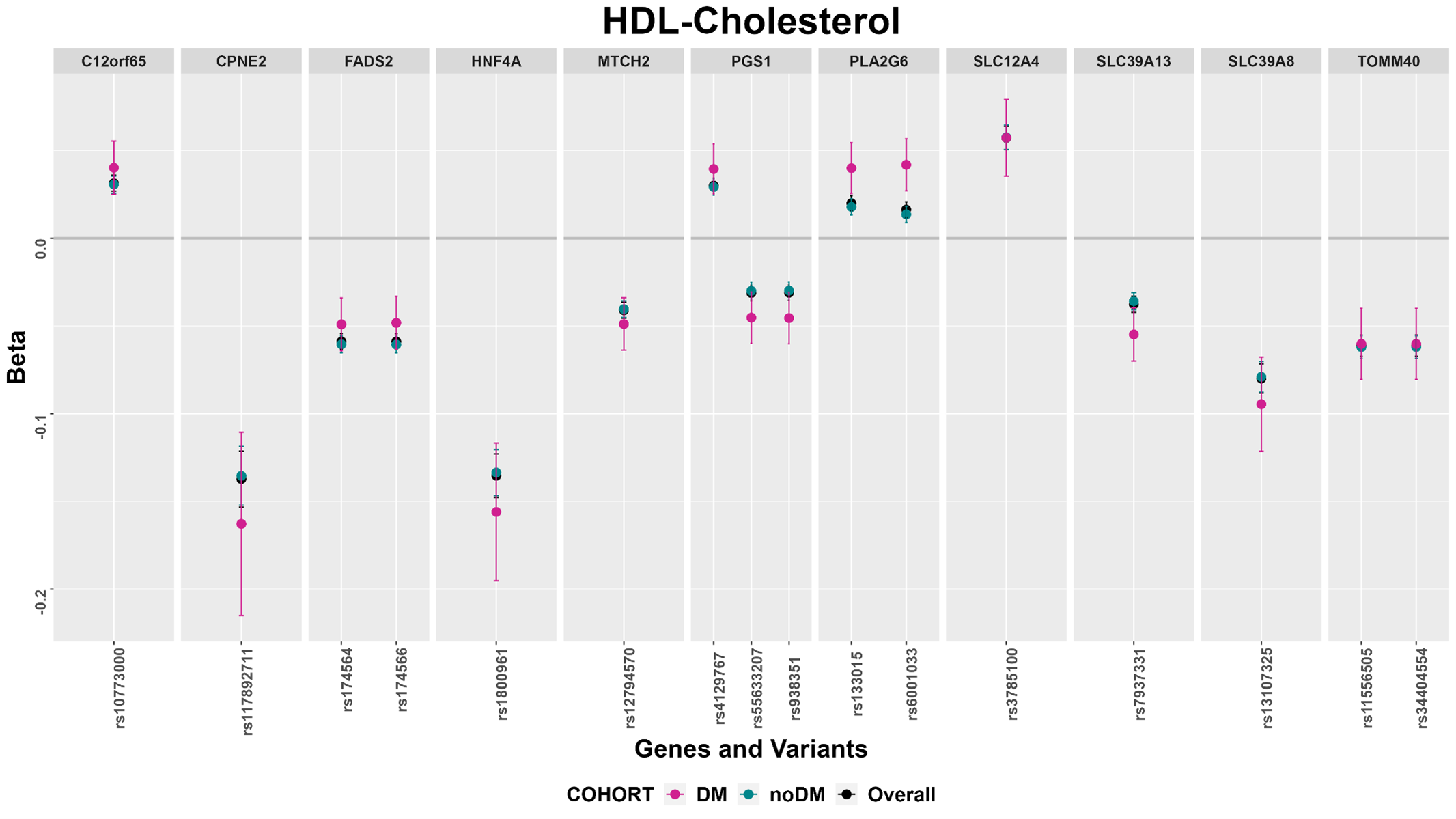

Supplement: Supplementary file 7 [file Image2.TIF]

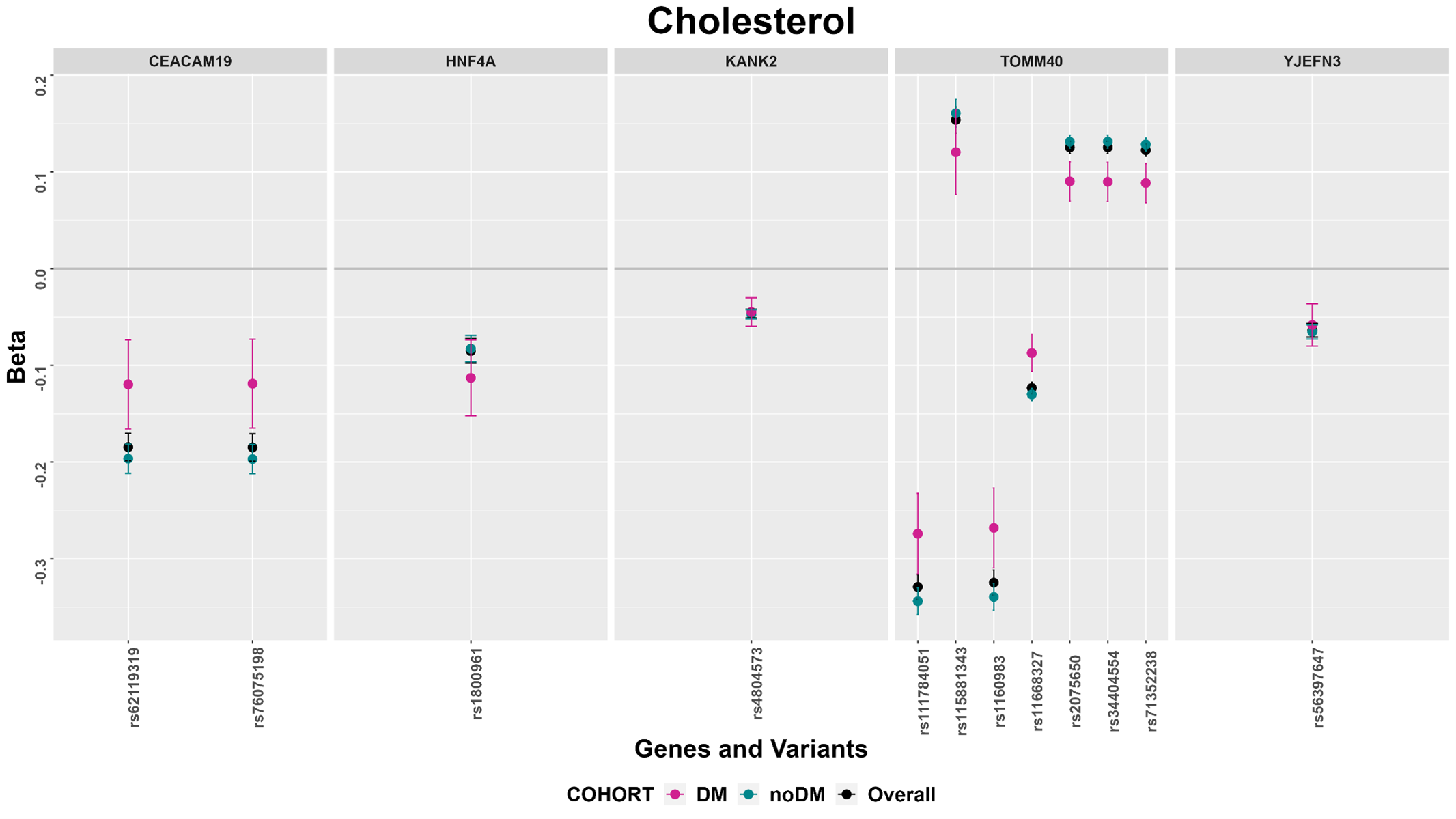

Supplement: Supplementary file 9 [file Image1.TIF]

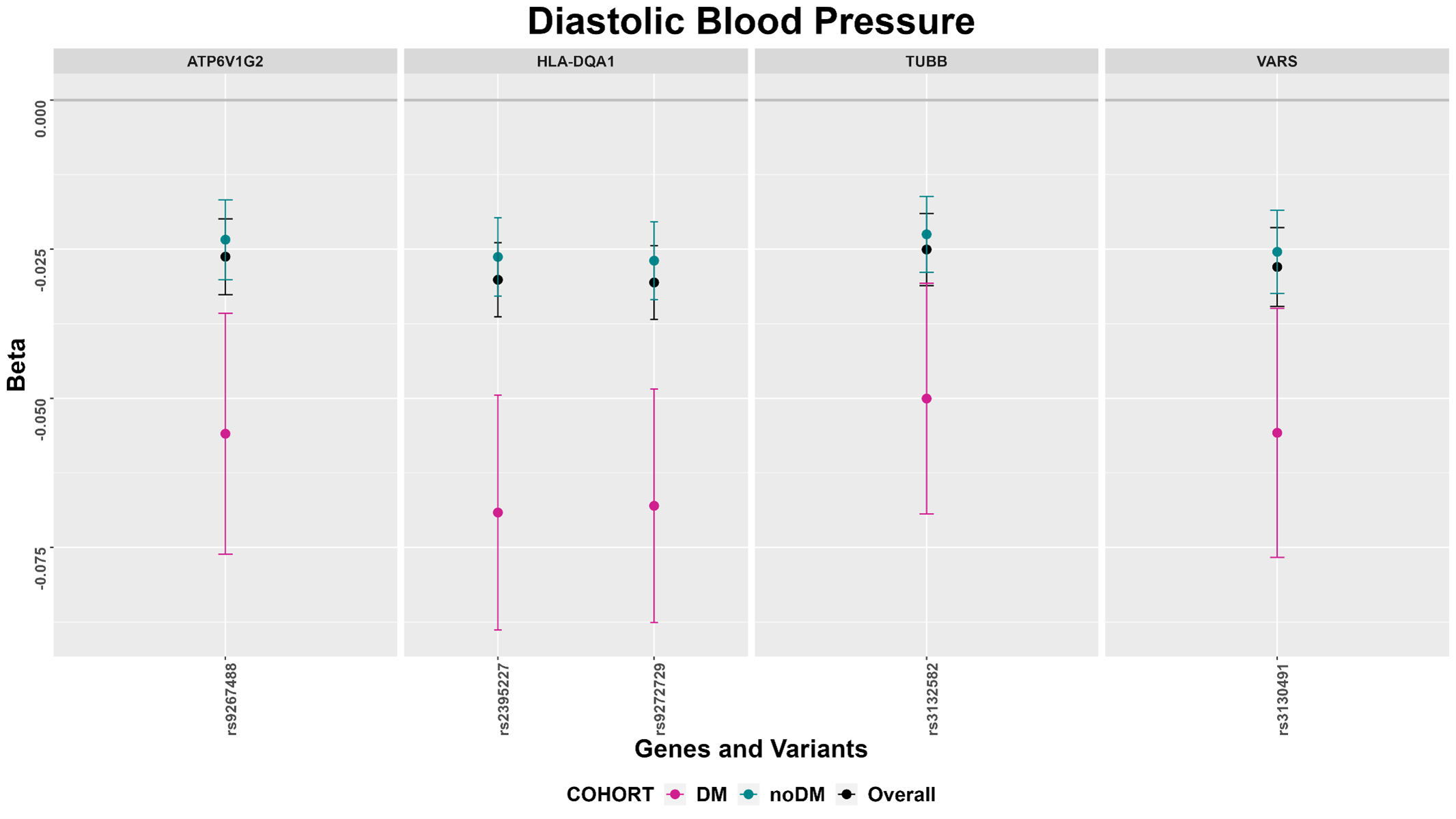

Supplement: Supplementary file 14 [file Image5.TIF]
