# Supplementary material for: Mitochondrial related variants associated with cardiovascular traits
Source: Front Physiol. 2024 Aug 27;15:1395371. doi: 10.3389/fphys.2024.1395371 (PMC11385366; doi:10.3389/fphys.2024.1395371)
Supplement: Supplementary file 16 [file DataSheet1.docx]

## Supplementary Methods

### Outcome Variables

Data fields used to calculate variables are listed in Supplementary Table 1.

#### Cardiovascular Disease

CVD was defined as the presence of any pathology, condition or procedure indicative of vascular disease, according to the information provided in the variables non-cancer illnesses, self-reported (Data field #20002), participants operations (Data field #20004), or medication impacting blood pressure in the variable treatment/medication (Data field #20003). Details can be found in Supplementary Table 2.

#### Coronary Artery Disease

Coronary Artery Disease (CAD) was defined as ‘heart attack/myocardial infarction’ (category 1075 in non-cancer illness, Data field #20002), operations (Data field #20004) including percutaneous transluminal coronary angioplasty (PTCA, category 1070), coronary artery bypass graft (CABG, category 1095) or triple heart bypass (category 1523) or ‘heart attack’ in vascular/heart problems diagnosed by doctor (Data field #6150), as previously defined by Inouye et al (Inouye et al., 2018b).

#### Blood Pressure Variables

##### Systolic and Diastolic Blood Pressure

Systolic blood pressure (SBP) and diastolic blood pressure (DBP) were calculated as the average of automatic measures (Data fields #4080 and #4079) or manual measures (Data fields #93 and #94) in its absence.

##### Hypertension

Participants were considered with hypertension when SBP ≥ 130 mmHg or DBP ≥ 80 mmHg (Stage 1 Hypertension), according to US and NICE guidelines (National Institute for Health and Care Excellence [NICE], 2017; Whelton et al., 2018; National Institute for Health and Care Excellenve (NICE), 2019); individuals with medication or conditions related to hypertension were also considered. Individuals with blood pressure medication (Data fields #6153 and #6177) or other related medications (Data field #20003; see Hypertensive Medication for more details), non-cancer illness (Data field #20002) and participants operations (Data field #20004) indicative of hypertension were considered. Details can be found in Supplementary Table 2.

#### Serum Variables

Serum variables Cholesterol (mmol/L), high-density lipoprotein (HDL) Cholesterol (mmol/L), low-density lipoprotein (LDL) Direct (mmol/L) and Triglycerides (mmol/L) were taken from Data fields #30690, #30760, #30780 and #30870, respectively.

### Other Variables (in alphabetical order)

#### ANTI-Hypertensive Medication

Participants were considered under anti-hypertensive medication if they had any record of anti-hypertensive drugs falling in the categories: angiotensin-converting enzyme (ACE) inhibitors (ACEi), angiotensin II receptor blockers (ARBs), diuretics, calcium channel blockers, beta blockers, alpha blockers and combination drugs from these categories, identified in Data field #20003 (Treatment/medication code). Anti-platelet drugs, anticoagulants, anti-angina meds, anti-arrhythmic agents, statins and medicines for erectile dysfunction or prostate enlargement (which have hypotensive actions) were excluded, since they are not used directly for management for hypertension. Participants were also considered under anti-hypertensive medication if they had the category “Blood pressure medication” in Data Fields #6577 (Medication for cholesterol, blood pressure or diabetes) or #6153 (Medication for cholesterol, blood pressure, diabetes, or take exogenous hormones). Full list of medications used is available on request to the corresponding author.

#### Cholesterol Medication

Participants were considered under cholesterol treatment when had the category “Cholesterol lowering medication” in Data Fields #6577 (Medication for cholesterol, blood pressure or diabetes) or #6153 (Medication for cholesterol, blood pressure, diabetes, or take exogenous hormones), or had the Treatment/medication codes 1140861958 (simvastatin), 1140888594 (fluvastatin), 1140888648 (pravastatin), 1140910632 (eptastatin), 1140910654 (velastatin), 1141146234 (atorvastatin) or 1141192410 (rosuvastatin) in Data field #20003.

#### Diabetes

Participants were considered with diabetes when glycated hemoglobin ≥ 48 mmol mol^-1^ or blood glucose ≥ 7 mmol L^-1^ for participants with more than 8 fasting hours; individuals whose diabetes was diagnosed by a doctor (Data field #2443) were also considered. Other medications (Data field #20003), non-cancer illness (Data field #20002) and participants operations (Data field #20004) were considered to define diabetes. Details can be found in Supplementary Table 2.

#### Insulin

Participants were considered under insulin treatment when had the category “Insulin” in Data Fields #6577 (Medication for cholesterol, blood pressure or diabetes) or #6153 (Medication for cholesterol, blood pressure, diabetes, or take exogenous hormones), or had the Treatment/medication code 1140883066 (insulin product) in Data field #20003.
